# Efficient and safe lung gene delivery using AAV6.2FF in neonatal pigs demonstrates pediatric translational potential

**DOI:** 10.1016/j.omta.2026.201739

**Published:** 2026-05-14

**Authors:** Nicole Zielinska, Erin L. Howard, Cici Yang, Brenna A.Y. Stevens, Melanie M. Goens, Yanlong Pei, Brad Thompson, Jeff L. Caswell, Bernard Thebaud, Douglas Wey, Alexander Valverde, Luis G. Arroyo, Sarah K. Wootton

**Affiliations:** 1 Department of Pathobiology, University of Guelph, Guelph, ON N1G 2W1, Canada; 2 Avamab Pharma Inc., Calgary, AB T2T 2P9, Canada; 3 Ottawa Hospital Research Institute, Ottawa, ON K1Y 4E9, Canada; 4 Department of Animal Biosciences, University of Guelph, Guelph, ON N1G 2W1, Canada; 5 Department of Clinical Studies, Ontario Veterinary College, Guelph, ON N1G 2W1, Canada

**Keywords:** adeno-associated virus, AAV, AAV6.2FF, lung gene therapy, neonatal pigs, surfactant protein B deficiency, atomizer, AAV vector biodistribution

## Abstract

While adeno-associated virus (AAV) vectors have demonstrated efficacy in multiple organ systems, gene delivery to the lung remains an unmet therapeutic target. To treat a monogenic lung disease like surfactant protein B deficiency, a gene delivery system must overcome pulmonary barriers and be validated in models that closely resemble the human respiratory tract. Here, we optimized the endotracheal delivery of AAV6.2FF, an engineered capsid with enhanced pulmonary tropism, in neonatal piglets with lung features comparable to those of human newborns. We optimized delivery parameters that improved pulmonary distribution and applied them to administer AAV6.2FF expressing secreted alkaline phosphatase (SEAP) at two different doses: 5 × 10^12^ vg/kg and 1.73 × 10^13^ vg/kg to assess transgene expression, biodistribution, and the safety and tolerability of this AAV platform. Transgene expression was dose-dependent, with the higher dose producing more robust and widespread SEAP expression across the cranial, middle, accessory, and upper caudal lobe regions. Both doses were well tolerated, with low anti-AAV6.2FF IgG titers and no hematology and biochemistry markers showing trends indicative of systemic toxicity. This study establishes the neonatal pig as a clinically relevant model for optimizing lung-directed AAV dosing and delivery, while also highlighting the need for further optimization to maximize this platform’s therapeutic potential.

## Graphical abstract

**Figure:**
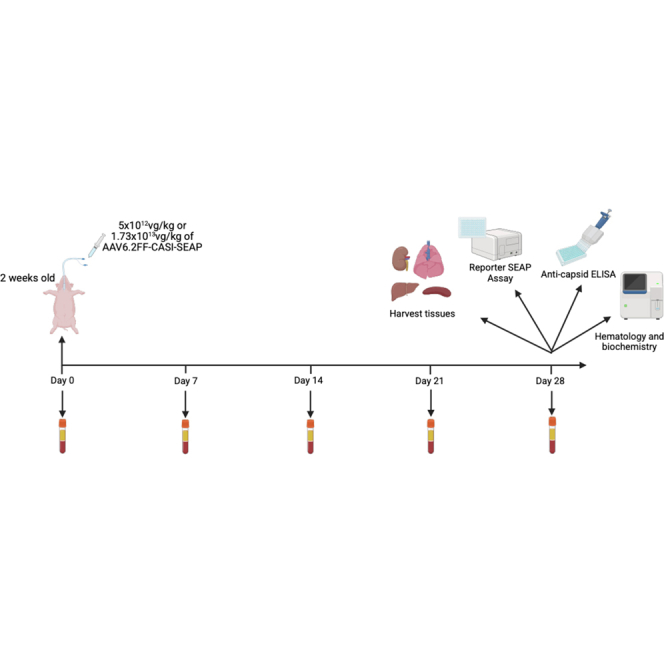

## Teaser

Zielinska and colleagues present a clinically relevant neonatal pig model to optimize AAV6.2FF-mediated lung gene delivery. Their findings identify administration refinements that improve pulmonary distribution, dose-dependent transgene expression and safety, advancing the translation of AAV-based therapies to treat fatal monogenic lung diseases.

## Introduction

Gene therapy has opened the door to treating inherited diseases that were once considered untreatable.^1^^,^^2^ Adeno-associated virus (AAV) vectors have played a significant role in these advancements, leading to approved treatments for inherited conditions affecting the eye, liver, muscle, and central nervous system.^2^^,^^3^^,^^4^^,^^5^ However, there are no approved AAV therapies for diseases involving the respiratory tract, due to the physical and immunological barriers that make targeting the lungs considerably more difficult.^6^^,^^7^^,^^8^^,^^9^^,^^10^^,^^11^ Adding to this challenge is the aspect of clinical translation. Although some studies in mice have shown effective AAV delivery to the lungs, results from mouse models do not always translate to larger animals and ultimately humans.^12^ Differences in lung size, airway structure, and immune response between species limit the relevance of murine data to larger animal applications,^13^^,^^14^^,^^15^ emphasizing the need to optimize lung gene delivery in large animal models that more accurately reflect human pulmonary anatomy and physiology.

In response to the physical and immunological barriers, researchers have worked to optimize AAV genomes, capsids, and administration techniques to improve gene delivery to the lungs.^14^^,^^16^^,^^17^^,^^18^ One novel capsid that emerged from this effort is AAV6.2FF. After modifying the AAV6 capsid to include three point mutations, our lab was able to enhance widespread lung transduction in mice following intranasal and intratracheal administration.^19^ Given these properties, AAV6.2FF has also been used preclinically in the treatment of surfactant protein B (SP-B) deficiency in mice.^20^^,^^21^

SP-B deficiency is a fatal monogenic lung disease caused by mutations within the SFTPB gene, which leads to respiratory failure within the first few days of life.^20^^,^^22^^,^^23^^,^^24^^,^^25^ Preclinical studies show the therapeutic potential of AAV6.2FF for treating SP-B deficiency in mice, where intratracheal administration of AAV6.2FF carrying the SP-B gene significantly improved lung function and ultimately prolonged survival.^20^ Despite these promising results in mice, they have yet to be replicated in a larger animal model that more accurately reflects the anatomical, physiological, and immunological complexity of the human lung. The US Food and Drug Administration (FDA) recommends conducting gene therapy studies in large-animal models when smaller species are unlikely to capture human anatomical or physiological complexity. This approach is consistent with regulatory guidance for translational safety and biodistribution assessment prior to clinical testing.^26^ An appropriate large animal model is the neonatal pig, as its lung size, airway structures, and innate immune responses closely mimic those of human infants.^27^^,^^28^^,^^29^^,^^30^ However, before AAV6.2FF can be considered for clinical use in SP-B deficiency and deliver the therapeutic human SPB (hSPB) gene, the dosing and method of administration must be optimized to ensure consistent and widespread lung transduction, as well as confirmation of safety and tolerability.

In this study, we used AAV6.2FF expressing a SEAP reporter gene to optimize lung gene delivery in a neonatal pig model and establish a translatable platform for SP-B deficiency. Because infants with SP-B deficiency are often intubated at birth,^31^^,^^32^ we delivered our vector endotracheally using a mucosal atomization device to simulate a clinically relevant approach and promote greater distribution in the lungs. We tested two different vector doses and assessed their influence on transgene expression and distribution. We also evaluated the safety, tolerability, and anti-capsid immune responses to better understand how the vector was tolerated in this pig model. Altogether, this work establishes effective administration strategies that will help translate this therapy to human infants in the future.

## Results

### Lung delivery optimization in a neonatal pig model

**Figure 1:**
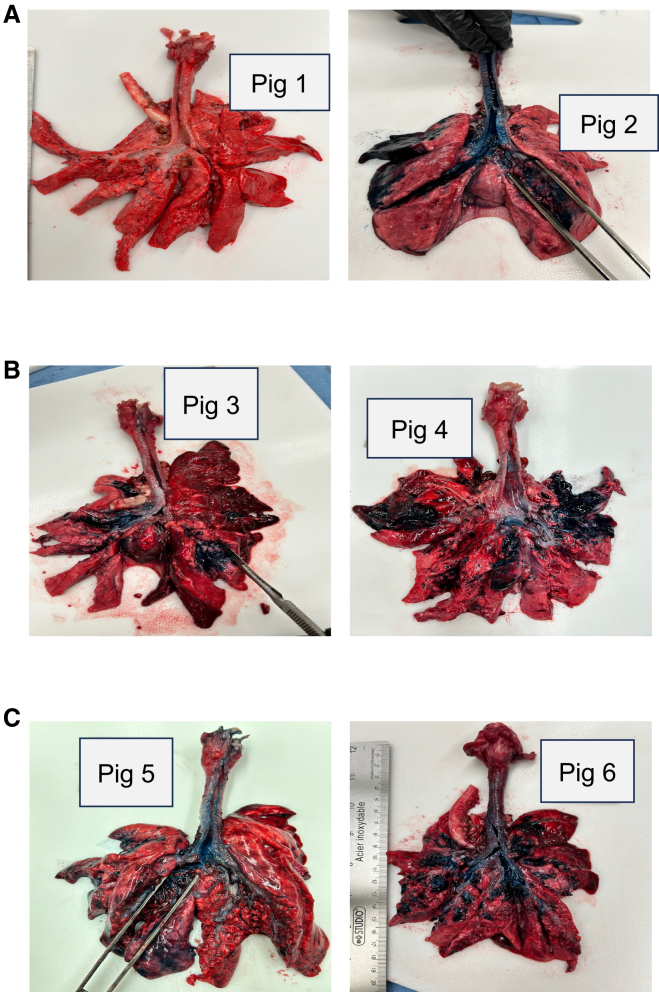
Optimization of lung delivery in a neonatal model Six piglets were sedated and administered 0.5 mL/kg of 3% Evans blue dye into the lungs using a MADgic laryngo-tracheal mucosal atomization device inserted through a laryngoscope into the trachea. Delivery conditions were changed across three studies to evaluate the factors that enhance dye distribution in the lungs. After their assigned recovery time on oxygen, piglets were euthanized, and lungs were excised and evaluated for Evans blue distribution throughout the airways and parenchyma. (A) The atomizer was inserted down the trachea until resistance was felt near the carina, and then the piglets were given 5 min of oxygen recovery in a prone, or stomach-down position. (B) The atomizer was placed more proximally in the trachea to promote a better mist when delivering the dye. Pigs were rolled gently side to side after administration and allowed 5 min of oxygen recovery. (C) The dye was administered from the same height as in (B), and the pigs were held upright for 2 min post-delivery to promote the dye to reach further into the lungs. These piglets were given a total of 10 min of oxygen recovery.

To determine the optimal delivery techniques that achieve the best aerosolized lung delivery in neonatal piglets, we performed a series of pilot studies using a MADgic laryngo-tracheal mucosal atomization device and 3% Evans blue dye. Each piglet received 0.5 mL/kg of dye intratracheally under varying procedural conditions. Following the delivery, piglets were allowed to recover for 30–45 min before euthanasia and lung excision to compare the degree of dye distribution in the lungs (Figure 1). In the first pilot study, the atomizer was inserted down the trachea until resistance was felt near the carina. The dye was administered, and the piglets were given 5 min of oxygen recovery in ventral recumbency (lying belly down) and monitored for an additional 30 min before being euthanized. In piglet 1, it is evident that the dye did not reach the lungs and was likely misdirected into the esophagus (Figure 1A). This was confirmed when we evaluated piglet 2, and proper tracheal entry was confirmed by palpating the tracheal rings with the atomizer device. Although the dye did reach the lungs of piglet 2, the staining was primarily restricted to one lung (Figure 1A). In the second study, the atomizer was inserted more proximally in the trachea to leave room to create the mist effect with the atomizer. Following delivery, we rolled the piglets from side to side during their 5-min oxygen recovery, and they were given an additional 40 min before euthanasia. In piglets 3 and 4, the dye reached multiple lobes but with more staining in the accessory lobe and the cranial and middle lobes of the lungs (Figure 1B). In the third pilot study, we used the same atomizer placement as we did in study 2. Following delivery, the piglets were held upright for 2 min to promote movement of the dye deeper into the lungs and the oxygen recovery was extended to 10 min. In piglets 5 and 6, the dye was visibly present in both sides of the lung and reached the caudal lobes with a more uniform distribution (Figure 1C). These results show that higher atomizer placement, upright positioning post-delivery, and extended oxygen recovery time improve lung distribution in a neonatal pig model. This optimized delivery approach was used in all subsequent *in vivo* experiments.

### Dose optimization of AAV6.2FF lung administration

To determine the dose needed for effective and widespread AAV delivery to the lungs, we administered AAV6.2FF-CASI-SEAP to neonatal piglets at either a low-dose (5 × 10^12^ vg/kg), or a high-dose (1.73 × 10^13^ vg/kg). Using our optimized delivery protocol, the piglets received 0.5 mL/kg of vector (diluted in PBS) endotracheally using an atomization device. At 28 days post-treatment, piglets were euthanized, and lungs were harvested *en bloc* for downstream analysis. To best visualize the biodistribution of transgene expression, lungs were divided into 16 predefined sampling regions labeled A through P (Figure S1). These regions account for both dorsal (Figure S1A) and ventral (Figure S1B) surfaces of the lung, enabling systematic mapping of AAV6.2FF-mediated SEAP expression. Multiple representative tissue samples were taken from each lung region and either fixed or stained for SEAP expression for macroscopic analysis or paraffin-embedded, sectioned and stained for SEAP expression for histological analysis.

**Figure 2:**
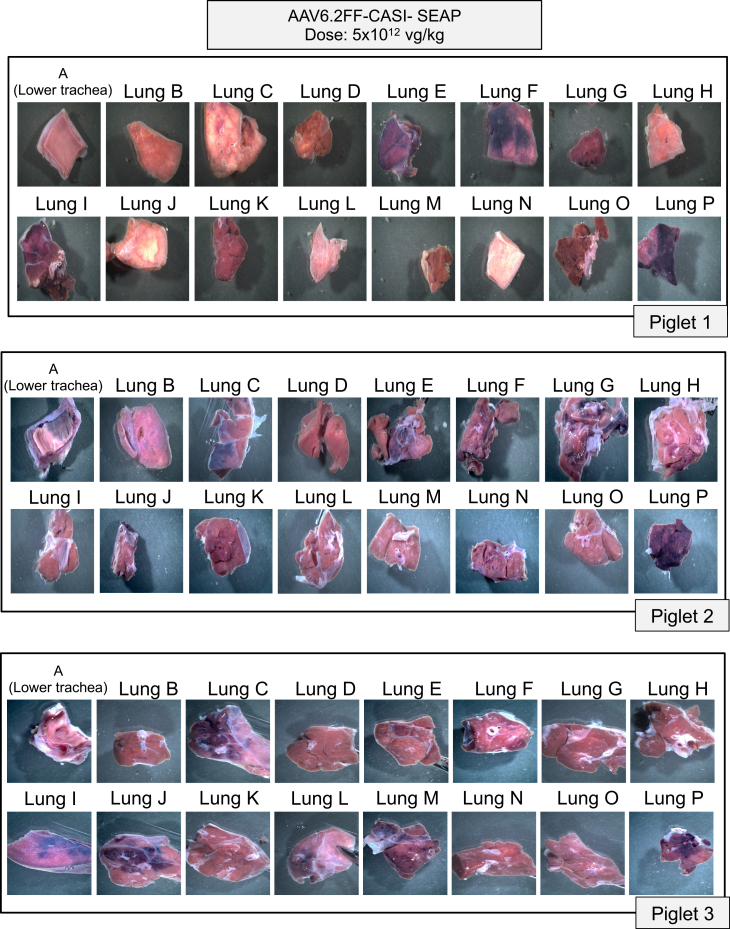
Macroscopic evaluation of alkaline phosphatase-stained lung sections following low-dose AAV6.2FF-CASI-SEAP administration in neonatal piglets About 2-week-old piglets (*n* = 3) were administered 5 × 10^12^ vg/kg of AAV6.2FF-CASI-SEAP via endotracheal atomization. After 28 days, piglets were euthanized, and the lungs were collected *en bloc*. Each lung was divided into 16 representative sampling regions (A-P), fixed, and stained for heat-stable alkaline phosphatase. Shown are representative macroscopic images of the stained lung sections from the three individual piglets.

**Figure 3:**
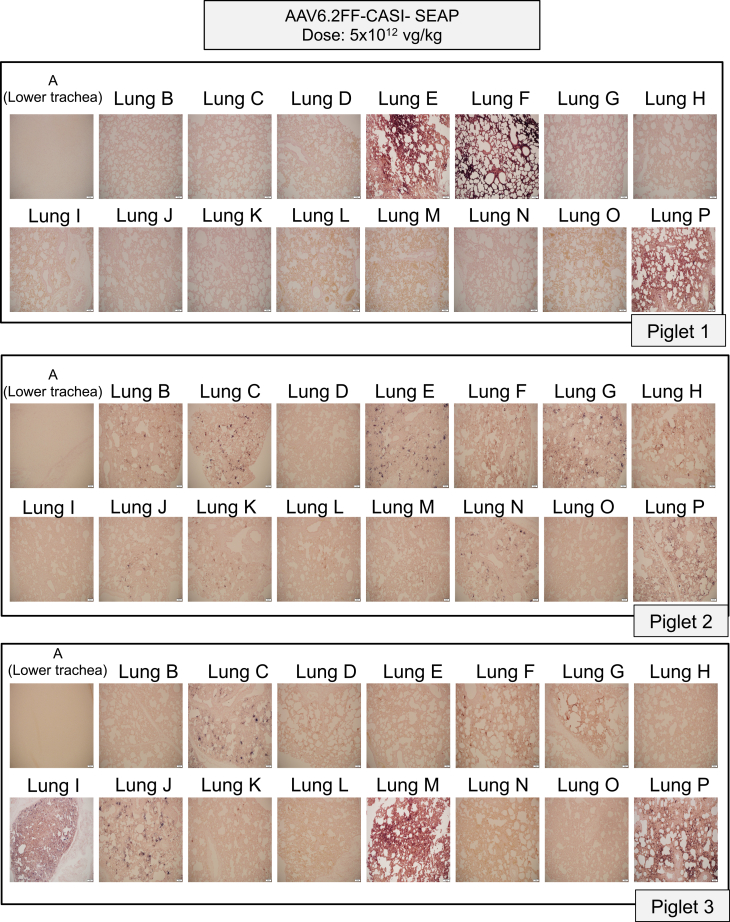
Histological evaluation of alkaline phosphatase-stained lung sections following low-dose AAV6.2FF-CASI-SEAP administration in neonatal piglets About 2-week-old piglets (*n* = 3) were administered 5 × 10^12^ vg/kg of AAV6.2FF-CASI-SEAP via endotracheal atomization and euthanized 28 days later. Lungs were excised, sectioned into 16 representative sampling regions (A-P), fixed, and stained for heat-stable alkaline phosphatase activity. Representative tissue sections from each region were paraffin-embedded, sectioned at 5 μm, and stained for alkaline phosphatase, followed by nuclear fast red counterstaining. Images were taken at 10× magnification (scale bars, 20 μm). Shown are the histological pictures from all 16 representative lung sections in each of the three piglets.

In the low-dose group, both macroscopic (Figure 2) and microscopic (Figure 3) staining showed limited SEAP expression in select regions of the lung with inter-animal variability. A commonality seen among the three piglets was that SEAP transduction was primarily confined to the cranial, middle, and accessory lobes with little expression observed in the caudal lobes (Figures 2 and 3). Interestingly, all three piglets showed SEAP expression in the accessory lobe found on the ventral side of the lungs, shown as section P (Figures 2 and 3). Although sections C and P from piglet 2 appeared adequately stained macroscopically, the histological analysis showed less pronounced SEAP expression at a cellular level (Figure 3). Rather than relying only on one representative image from each sampling site as shown in Figures 2 and 3, the entirety of every lung was subdivided into finer components before alkaline phosphatase (AP) staining to grossly visualize the overall pattern of transduction (Figures S2–S8).

**Figure 4:**
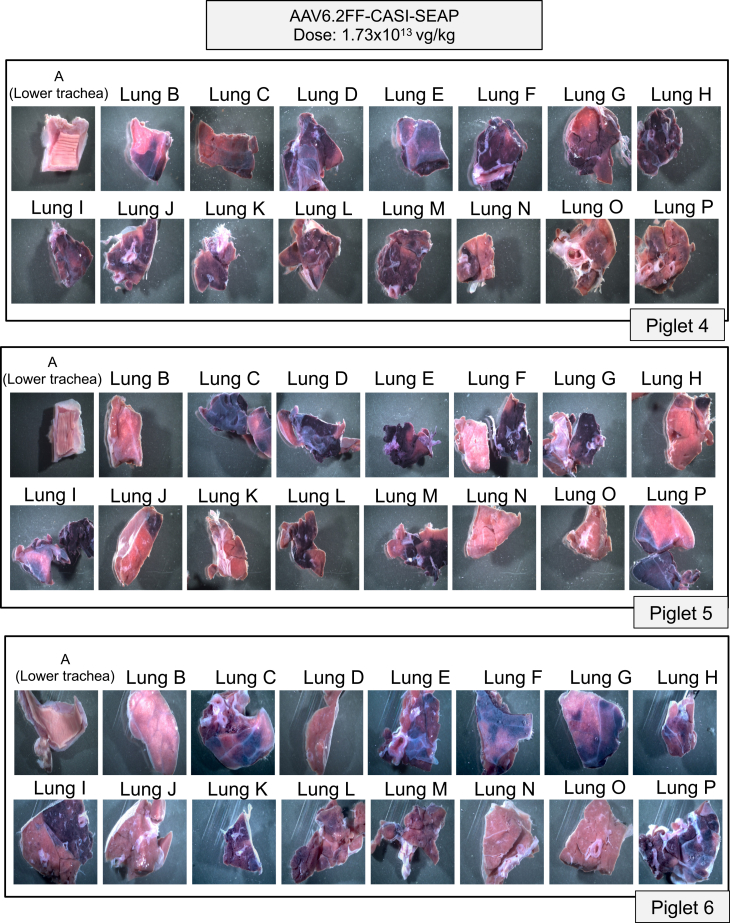
Macroscopic evaluation of alkaline phosphatase-stained lung sections following high-dose AAV6.2FF-CASI-SEAP administration in neonatal piglets About 2-week-old piglets (*n* = 3) were administered 1.73 × 10^13^ vg/kg of AAV6.2FF-CASI-SEAP via endotracheal atomization. After 28 days, piglets were euthanized, and the lungs were collected *en bloc*. Each lung was divided into 16 representative sampling regions (A-P), fixed, and stained for heat-stable alkaline phosphatase. Shown are representative macroscopic images of the stained lung sections from the three individual piglets.

**Figure 5:**
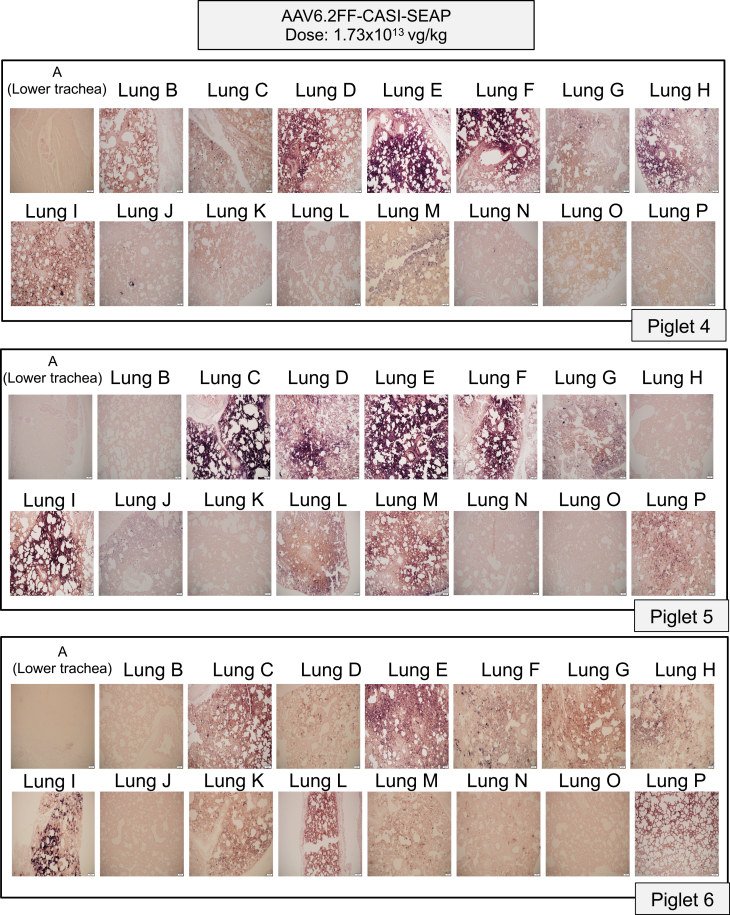
Histological evaluation of alkaline phosphatase-stained lung sections following high-dose AAV6.2FF-CASI-SEAP administration in neonatal piglets About 2-week-old piglets (*n* = 3) were administered 1.73 × 10^13^ vg/kg of AAV6.2FF-CASI-SEAP via endotracheal atomization and euthanized 28 days later. Lungs were excised, sectioned into 16 representative sampling regions (A-P), fixed and stained for heat-stable alkaline phosphatase activity. Representative tissue sections from each region were paraffin-embedded, sectioned at 5 μm, and stained for alkaline phosphatase, followed by nuclear fast red counterstain. Images were taken at 10× magnification (scale bars, 20 μm). Shown are the histological pictures from all 16 representative lung sections in each of the three piglets.

**Figure 6:**
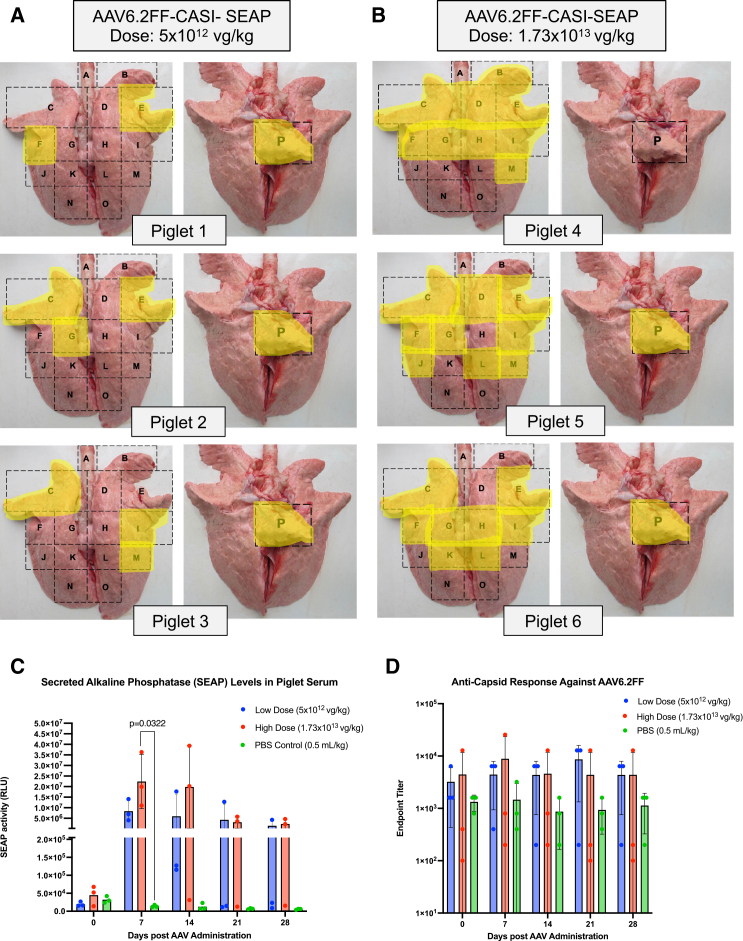
Dose-dependent pulmonary distribution and serum kinetics of SEAP expression and anti-AAV6.2FF antibody responses following intratracheal AAV6.2FF-CASI-SEAP delivery in neonatal piglets Two-week-old piglets (*n* = 3 per group) received either low-dose (5 × 10^12^ vg/kg), a high-dose (1.73 × 10^13^ vg/kg), or 0.5 mL/kg of PBS of AAV6.2FF-CASI-SEAP via endotracheal atomization. After 28 days, lungs were collected and analyzed for macroscopic and microscopic SEAP expression which was mapped (in yellow) on template lungs in dorsal and ventral views for the low-dose (A) and the high-dose (B). Serum was collected on days 0, 7, 14, 21, and 28 to assess (C) SEAP activity by luminescent reporter assay and (D) anti-AAV6.2FF capsid IgG titers by ELISA, reported as reciprocal endpoint titers. Data are shown as group means and error bars represent the standard deviation. Comparisons between the treatment groups at each time point were performed using a one-way ANOVA with Tukey’s post hoc test. Template lungs were adapted from Establishment of a Model of Mycoplasma hyopneumoniae infection using Bama miniature pigs by Gan et al. (https://doi.org/10.1186/s43014-020-00034-w) used under https://creativecommons.org/licenses/by/4.0/. Images were modified by adding region labels and highlighting.

In the high-dose cohort, we observed much stronger and widespread expression throughout the lungs of all the three piglets, both macroscopically (Figure 4) and microscopically (Figure 5). The pattern of distribution was much more consistent between replicates, with the vector repeatedly transducing regions in the cranial, middle, and accessory lobes as well as the cranial region of the caudal lobes. As seen in the low-dose group, the accessory lobe (section P) showed robust transduction for piglets 5 and 6 (Figure 5). Notably, expression did not extend into regions N and O, which are the most caudal areas of the right and left caudal lobes (Figure 5). By combining the results from the macroscopic, microscopic and subdivided tissue staining analyses from all piglets across both doses, we generated composite lung maps for each piglet to summarize the anatomical distribution of transduction across all 16 lung regions (Figures 6A and 6B). Subdivided tissue staining analyses (Figures S4–S6) and mapping of the SEAP-positive regions onto a composite map (Figures 6A and 6B) illustrate the improved degree of transgene distribution in the lungs compared to the low-dose and PBS-treated groups (Figures S9 and S10).

High-magnification analysis of SEAP-stained lung sections revealed robust transgene expression within alveolar regions, with minimal to no expression detected in bronchial airway epithelial cells (Figure S14). This distribution is consistent with preferential alveolar transduction and further supports the observed epithelial targeting of the AAV6.2FF vector in the pig lung.

To clarify the lung cell types transduced by the AAV6.2FF vector in the pig model, we employed multiple complementary approaches. Immunohistochemical (IHC) analyses demonstrated co-localization of secreted alkaline phosphatase (SEAP) expression with thyroid transcription factor-1 (TTF-1), a well-established marker of pulmonary epithelial cells, supporting epithelial cell transduction by AAV6.2FF (Figure S15). Attempts to further characterize transduced cells using immunofluorescence were limited by substantial tissue autofluorescence, which precluded reliable co-staining of SEAP-expressing cells.

To further interrogate cell identity, RNAscope *in situ* hybridization was evaluated. The assay was confirmed to be functional in fresh pig lung tissue that was harvested, fixed, and processed immediately, as evidenced by robust detection of a housekeeping gene (Figure S16). In contrast, lung tissues subjected to heat inactivation and histochemical staining for SEAP were not amenable to RNAscope analysis, presumably due to RNA degradation resulting from these processing steps. Consequently, RNAscope could not be used to definitively identify the transduced cell populations in these samples.

To determine whether increasing the vector dose influenced off-target expression, we examined several non-pulmonary tissues for SEAP activity. The heart, liver, kidneys, spleen, and proximal trachea were collected from all piglets and processed for both macroscopic (Figure S11) and histological (Figure S12) SEAP staining. Across both doses and PBS control groups, SEAP transduction was undetectable in the heart, liver, spleen, and kidneys. Mild SEAP staining was observed in the proximal trachea of treated piglets, which was expected due to the endotracheal (ET) route of vector administration (Figure S12). Overall, these results support the specificity of our vector delivery regardless of vector dose.

### Kinetics of transgene expression and immune responses

To monitor the kinetics of circulating transgene expression and anti-AAV6.2FF capsid immune responses following AAV6.2FF-CASI-SEAP administration, blood samples were collected on days 0, 7, 14, 21, and 28 post AAV. Circulating SEAP activity (relative light units; RLU) was quantified using a luminescent SEAP assay (Figure 6C), and anti-capsid IgG endpoint titers were evaluated by ELISA (Figure 6D). As expected, SEAP activity was minimal at day 0 since this time point corresponds to the pre-bleeds collected prior to AAV administration (Figure 6C). These values are background SEAP levels from untreated animals. By day 7, circulating SEAP expression reached the highest levels in both the low-dose and the high-dose groups seen across all time points with means of 8.36e6 RLU and 2.24e7 RLU, respectively (Figure 6C). By day 14, groups maintained substantial SEAP levels of 5.98e6 RLU and 1.98e7 RLU, respectively, with a modest decrease seen at day 28 of 1.43e6 RLU and 2.30e6 RLU, respectively. The only statistically significant difference was at day 7 between the high-dose and PBS control (*p* = 0.0322). These data shows that lung administration of AAV6.2FF-CASI-SEAP led to high enough SEAP expression to be detected in the blood following intratracheal administration, with peak transgene levels observed between days 7 and 14.

To test the immune response against the AAV.2FF capsid, ELISA plates were first coated with AAV6.2FF particles and incubated with collected serum samples from all piglets and time points. An HRP-conjugated anti-porcine IgG secondary antibody was added to bind any pig IgG that attached to the AAV capsid. The amount of bound antibody was measured using a colorimetric substrate reaction. The endpoint titers were defined as the highest dilution of serum that produced an optimal density (OD) value at least twice that of the negative control. Across all the time points, antibody titers remained relatively low in all groups, including those treated with both doses of AAV (Figure 6D). Most PBS-treated piglets had endpoint titers below 1 × 10^3^, while AAV-treated piglets had modest increases with titers ranging between 1 × 10^3^ and 1 × 10^4^ (Figure 6D). These elevated titers were not universal across all treated piglets, and titers fluctuated over time without a clear pattern (Figure 6D). Most importantly, there was no indication of a dose-dependent relationship, as both low- and high-dose treated groups showed similar and variable titers across the study.

### Safety and tolerability of AAV6.2FF in a neonatal pig model

**Figure 7:**
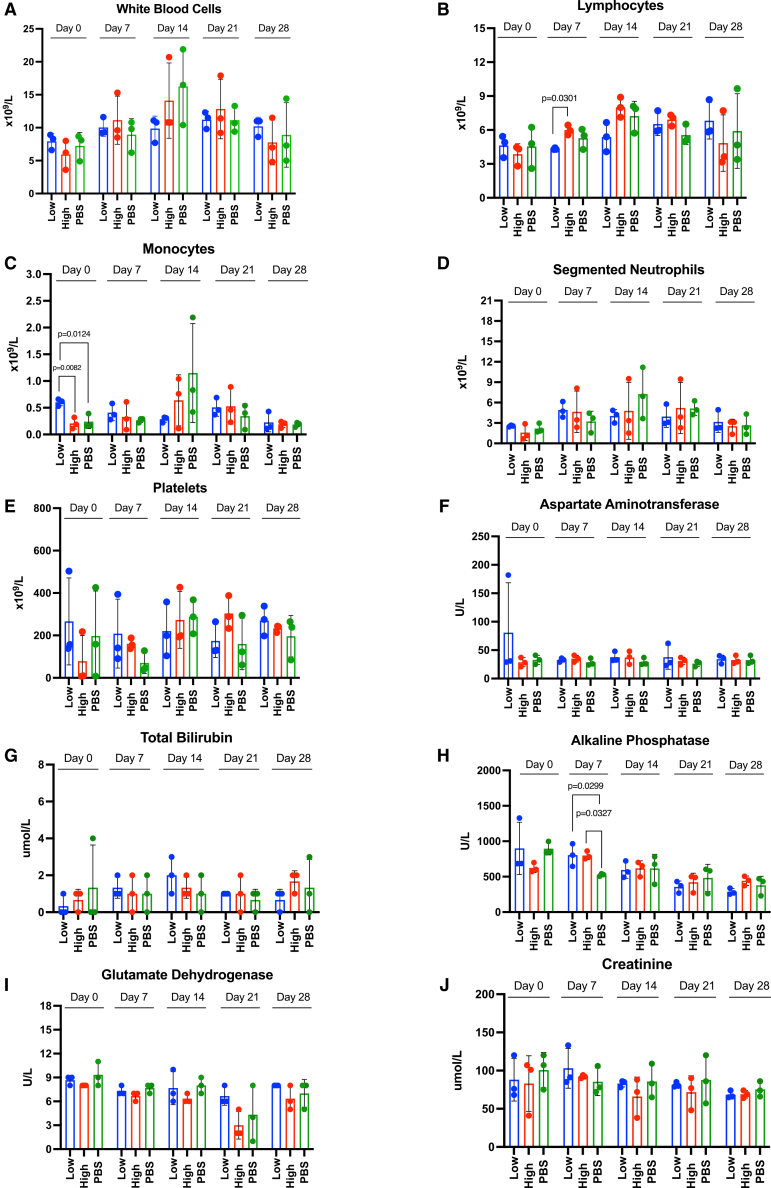
Safety and tolerability assessment of AAV6.2FF-CASI-SEAP administration via hematology and biochemistry profiling Two-week-old piglets were administered either a low dose (5 × 10^12^ vg/kg) of AAV6.2FF-CASI-SEAP (*n* = 3), a high-dose (1.73 × 10^13^ vg/kg) (*n* = 3), or 0.5 mL/kg (*n* = 3) of PBS. Blood was collected from piglets at days 0, 7, 14, 21, and 28 post-treatments for hematological (A) white blood cells, (B) lymphocytes, (C) monocytes, (D) segmented neutrophils, (E) platelets, and biochemistry analysis (F) aspartate aminotransferase, (G) total bilirubin (H) alkaline phosphatase, (I) glutamate dehydrogenase, and (J) creatinine. Mean values are shown on all graphs, with error bars representing the standard deviation (SD). A one-way ANOVA with Tukey’s post hoc test was used to assess significance between treatment groups at each time point.

To test the safety and tolerability of endotracheally administered AAV6.2FF-CASI-SEAP in neonatal piglets, peripheral blood was collected on days 0, 7, 14, 21, and 28 and analyzed for hematological and biochemistry markers (Figure 7; Figure S13). Piglets received either a low-dose (5 × 10^12^ vg/kg), a high-dose (1.73 × 10^13^ vg/kg), or PBS as a control. Complete blood counts (CBCs) largely remained within the expected reference intervals, and there were no dose-dependent changes that indicated acute toxicity. White blood cell counts (Figure 7A), segmented neutrophils (Figure 7D), and platelet levels (Figure 7E) showed no notable differences over time. Monocytes (Figure 7C) showed a statistically significant difference at the pre-bleed (day 0) between the low-dose and both the PBS (*p* = 0.0124) and the high-dose (*p* = 0.0082) groups. However, these levels were within the normal reference interval and were unrelated to AAV treatment because the blood was collected prior to AAV administration. Similarly, lymphocyte counts (Figure 7B) showed an increase in the high-dose at day 7 compared to the low-dose (*p*=0.0301) but these levels were within the normal interval range, and this difference was not seen at later time points.

Biochemical markers aspartate aminotransferase (Figure 7F), glutamate dehydrogenase (Figure 7I) and total bilirubin (Figure 7G) remained within normal reference intervals across all groups. Alkaline phosphatase (ALP) levels did exceed the upper reference limit (500 U/L), with statistically significant increases seen between both AAV-treated groups compared to the PBS group at day 7 (Figure 7H). However, these levels were transient, and by day 14, AAV-treated animals had similar levels of ALP levels to the PBS-treated animals. Supplemental analyses on additional hematological and biochemistry markers also support that AAV6.2FF-CASI-SEAP administration was well tolerated (Figure S13).

## Discussion

Developing a successful gene therapy for monogenic lung diseases requires a delivery platform to be both effective and clinically relevant to humans. Although AAV6.2FF exhibits strong pulmonary tropism in mice, vector tropism, and promoter activity can vary substantially across species due to differences in airway architecture, glycans, and host transcriptional machinery.^33^^,^^34^^,^^35^ The neonatal pig provides a valuable intermediary model because its lung physiology, airway branching, and epithelial composition closely resemble those of human infants, offering a more predictive platform for evaluating AAV6.2FF-mediated gene transfer in pediatric lungs.^29^^,^^36^ Here, we optimized the ET delivery of AAV6.2FF in a neonatal pig model to establish a relevant platform that could one day be applied for the treatment of SP-B deficiency. We identified key delivery techniques that enhance the widespread pulmonary distribution of AAV6.2FF and evaluated two vector doses to determine how they influence transgene expression, lung distribution, and the safety and tolerability of this gene therapy.

Many ET delivery strategies have been used in both clinical and research settings including bolus instillation through an ET tube, nebulization, and aerosolization.^37^^,^^38^^,^^39^^,^^40^^,^^41^ Direct instillation via ET tube is the most common method for surfactant replacement therapy in infants with respiratory distress syndrome or SP-B deficiency.^42^^,^^43^ While this method delivers a dose quickly to the lungs, it does so in the form of a liquid, which can lead to uneven distribution because of gravitational pooling.^44^ Nebulizers offer an alternative to ET tubes by producing aerosolized droplets that enable finer dispersion of these droplets in the lungs.^45^^,^^46^^,^^47^^,^^48^ However, nebulizers often require extended delivery times, are prone to much more vector loss and typically require specialized equipment.^46^^,^^47^^,^^48^ To overcome these challenges, we chose a laryngo-mucosal atomization device to deliver AAV6.2FF-CASI-SEAP as a fine spray. This atomizer enables direct airway administration while producing a fine mist (30–100 μm).^49^^,^^50^^,^^51^

Through the refinement of our delivery technique, we identified three important delivery modifications that dramatically improve the distribution of the vector to the lungs: (1) positioning the atomizer more proximally in the trachea, (2) holding the piglets in an upright position (head up) after administration, and (3) extending the duration of oxygen recovery. Placing the atomizer more proximally in the trachea allowed for enough distance from the bifurcation to create an aerosol mist upon release, resulting in finer droplets that could be more easily distributed throughout the lungs. By holding the piglets in an upright position after delivery, gravity likely helped guide the vector deeper into the lungs. Lastly, increasing the oxygen recovery time likely stabilized the piglets breathing and prevented coughing. When combined, these three modifications helped develop an efficient and reproducible approach for delivering aerosolized liquids.

The level of transgene expression we saw between the low-dose (5 × 10^12^ vg/kg) and high-dose (1.73 × 10^13^ vg/kg) AAV6.2FF-CASI-SEAP groups appeared to be clearly dose dependent. In the high-dose group, SEAP expression was repeatedly observed in the cranial, middle, and accessory lobes as well as the cranial regions of the caudal lobes, while the low-dose group showed limited expression restricted to the cranial, middle, and accessory lobes. Importantly, there was no SEAP expression detected macroscopically or microscopically in non-pulmonary tissues, including the heart, liver, kidneys, and spleen. This supports previous studies showing that AAV6.2FF has preferential tropism to the lungs with limited off-target expression when delivered intratracheally.^52^ Although the high-dose improved widespread transduction relative to the low-dose, it did not reach the most caudal parts of the lungs (sections N and O), leaving room for improvement. Phase 1/2 studies of aerosolized AAV for cystic fibrosis in adults have used doses as high as 2 × 10^15^ vg per patient suggesting that our current dose may still be sub-therapeutic for maximal effect.^53^ Future studies should explore the effects of higher doses and delivery volumes, as well as the co-administration of vector with bovine lipid extract surfactant (BLES)^54^^,^^55^ or formulation in 3%–7% sodium chloride (NaCl),^56^^,^^57^^,^^58^^,^^59^ which have been previously shown to enhance distribution and transduction, respectively.

Serum SEAP expression was higher in the high-dose group at every time point but only reached statistical significance at day 7 when compared to the other groups. Although serum anti-AAV6.2FF IgG titers were relatively low in all groups; they varied between individual piglets with no clear dose-dependent pattern, suggesting a degree of individual immune response. While the higher vector dose may have transiently increased transgene expression, it also could have triggered a gradual adaptive immune response, resulting in some vector clearance. Future work should evaluate neutralizing antibody titers to better understand how immune responses might limit the use of higher doses and the overall long-term efficacy.

All the cohorts of piglets showed tolerance to the two doses of AAV6.2FF-CASI-SEAP. Across all hematology and clinical markers, piglets largely remained within swine-specific reference intervals and showed no trends that would indicate systemic toxicity.^60^ A few occasional statistical changes were observed, for example, the AP activity for both vector groups at day 7. This elevation is likely attributed to the exogenous expression from the heat-stable AP reporter gene delivered by our AAV6.2FF vector rather than liver injury, as other hepatic enzymes and biomarkers remained within expected swine reference intervals and showed no dose-dependent changes. In addition, AP activity was already elevated in piglets at day 0 before any AAV was administered and then gradually decreased after day 14, suggesting this increase was unrelated to the vector and more likely due to age-related physiological variation, a known phenomenon in growing piglets.^61^^,^^62^ There were also statistically significant differences in monocytes at day 0 and lymphocytes at day 7; however, both values remained within the normal ranges^60^ and were not sustained over time. All together, these levels support the safety profile of AAV6.2FF-CASI-SEAP in this model.

While this study gave us important insights into optimizing lung gene delivery using AAV6.2FF in a neonatal model, the associated limitations should be taken into consideration. First, the small cohort size limited the statistical power for us to detect subtle effects that could help us understand the variability we saw in SEAP activity and anti-capsid data. Secondly, co-localization by standard chromogenic immunohistochemistry was technically limited because SEAP histochemical staining was highly intense and widespread, producing a saturated precipitate that obscures color contrast and prevents reliable multiplex color separation with cell-type markers. Future work will use multiplex immunofluorescence with an anti-SEAP antibody together with AT2 and additional epithelial markers to enable spectral separation or perform flow cytometry on dissociated lung with intracellular SEAP staining and AT2 markers to quantify transduced cell subsets. In addition, our study endpoint was capped at 28 days post-delivery and did not fully capture the duration of transgene expression, vector persistence, and late-onset immune responses that could affect the long-term safety and efficacy of this model and would therefore warrant additional studies that are carried out for a minimum of 6 months. Lastly, while we improved transgene distribution throughout the lungs using the high-dose of AAV, SEAP expression was still absent from the most distal parts of the lungs. As mentioned before, additional refinements are required to maximize the dispersion of the vector in the airway epithelium and explore other atomizers that produce a smaller droplet size.

In summary, this study establishes a clinically relevant pig model for evaluating AAV6.2FF-based lung gene delivery. We identified important administration strategies that improved vector delivery, demonstrated dose-dependent transgene expression, and confirmed the safety and tolerability of AAV6.2FF-CASI-SEAP at both tested doses. Together, these findings move us a step closer toward developing a meaningful gene therapy for rare lung diseases, like SP-B deficiency. Building on this work, future studies should explore whether higher doses and co-formulations with surfactant or NaCl improve distal lung targeting and observe long-term expression and immunogenicity by extending monitoring periods.

## Methods

### AAV vector production and titration

The AAV genome plasmid was engineered to encode a strong CASI promoter (comprised of the cytomegalovirus (CMV) enhancer, the chimeric chicken-β-actin (CAG) promoter and a ubiquitin C (UBC) enhancer region), the SEAP reporter gene, a woodchuck hepatitis virus post-transcriptional regulatory element (WPRE), and a simian virus 40 polyadenylation (SV40) sequence downstream of the SEAP transgene. The entire cassette was flanked by AAV2 inverted terminal repeats (ITRs) that facilitate packaging into the AAV6.2FF capsid. AAV6.2FF vectors were produced by co-transfecting adherent human embryonic kidney (HEK) 293 cells with the AAV genome plasmid and the pGDM6.2FF packaging plasmid and later purified using heparin affinity chromatography following a previously established protocol.^63^ The purified viral DNA was extracted using the Qiagen Blood and Tissue Kit (QIAGEN, Germantown, MD, 69504, USA), and genome titers were determined by TaqMan quantitative polymerase chain reaction (qPCR) using a TaqMan primer and probe set against the SV40 polyA signal (Integrated DNA Technologies), and the Luna universal qPCR master mix (New England Biolabs, Ipswich, MA, E1555L, USA) as previously described.^63^ Amplification and fluorescence detection were carried out using a LightCycler 480 thermocycler (Roche, Nutly, NJ, USA).

### Quality control

SDS-PAGE Coomassie blue gels were run to assess capsid protein ratios and size as well as purity of each vector preparation before pooling. Around 6%–15% gradient SDS-PAGE gels were made, and 4 × 10^10^ vg of each sample were loaded per lane. Samples were mixed with 4× reducing SDS buffer (Sigma-Aldrich, Oakville, Canada) and water for a final concentration of 1×, denatured at 95°C for 5 min, and run at 80 V for the first 20 min, then increased to 120 V for an additional 45 min. Gels were stained in 0.1% Coomassie Brilliant Blue R-250 staining solution prepared in 50% methanol and 10% glacial acetic acid for 20 min on the shaker. Gels were destained overnight in 40% methanol and 10% glacial acetic acid and imaged using a Bio-Rad ChemiDoc XRS system (Bio-Rad, Hercules, CA, USA). Alkaline agarose gels were run to visualize the AAV genomes to confirm size and identify any potential fragmentation. Around 1% agarose gels were prepared and soaked in 1× alkaline denaturing buffer (0.05 M NaOH, 1 mM EDTA) for 30 min 2 × 10^10^ vg of each vector was mixed with 2× denaturing loading dye (2× Ficoll loading Dye-50655, 1 M NaOH, 2 mM EDTA, and 0.6% SDS) and denatured at 95°C for 10 min. Samples were then loaded onto the 1% agarose gel and run at 40 V for 15 h at 4°C in 1× denaturing buffer. The gel was then soaked in 1× TAE buffer for 30 min and stained with SYBR Gold nucleic acid stain (Thermo Fisher Scientific, Waltham, MA, S11494, USA), prepared in 1× TAE buffer for 45 min and imaged using a UV transilluminator.

### Animal experiments: Optimization of ET delivery using 3% Evans dye

Two-week-old piglets (*n* = 6) were sedated using 5% isoflurane in 100% oxygen delivered through a coaxial non-rebreathing circuit via facemask until an appropriate level of anesthesia was achieved that allowed the introduction of the “atomizer probe” intratracheally. A laryngoscope was used to visualize the trachea, and a MADgic laryngo-tracheal mucosal atomization device (Teleflex, Wayne, PA, MAD600, USA) was guided down the laryngoscope into the trachea. About 3% Evans blue dye (Thermo Fisher Scientific, Waltham, MA, 45-000-059, USA) prepared in 1× PBS was administered at a volume of 0.5 mL/kg followed by a 1 mL of air chase. After delivery, pigs were held in an upright position sitting position with their ischial tuberosities in contact with a table for 2 min to facilitate gravity-assisted distribution into the distal lungs while maintained on oxygen. Pigs recovered for 10 min on oxygen and were monitored closely for an additional 30–45 min to assess respiration and general condition. Piglets were then sedated via intramuscular (i.m.) injection of dexmedetomidine (20–40 μg/kg), ketamine (10 mg/kg), and butorphanol (0.2 mg/kg), and blood samples (∼1 mL) were collected from the jugular vein. The piglets were then euthanized by intracardiac pentobarbital injection (0.3 mL/kg). The lungs were immediately excised and grossly examined for Evans dye distribution throughout the airways and parenchyma.

### Animal experiments: Dose optimization using AAV6.2FF

Two-week-old piglets (*n* = 9) were assigned to one of two dosing conditions. Each group had three piglets receiving 0.5 mL/kg of AAV6.2FF-SEAP at either 5 or 1.73 e13vg/kg. Three negative control piglets received 0.5 mL/kg of 1× PBS. For the vector administration and pre-bleed, we followed the same approach listed above, with the exception that animals were not euthanized post-AAV delivery. Instead, pigs were monitored for 28 days, and blood samples were collected retro-orbitally on days 7, 14, 21, and 28 post-treatment. On day 28, the piglets were sedated via i.m. injection of dexmedetomidine (20–40 μg/kg), ketamine (10 mg/kg), and butorphanol (0.2 mg/kg) and then euthanized by an intracardiac pentobarbital injection (0.3 mL/kg). The lungs were removed *en bloc* and divided into 16 labeled sections (A-P) (Figure S1). Each of these sections was further subdivided and allocated for either macroscopic or microscopic SEAP analysis. Major organs, including the heart, liver, spleen, kidneys, and higher trachea, were also collected and sectioned for parallel evaluation.

### Macroscopic analysis of SEAP expression

Representative sections of each lung subsection (A-P) and major organs were fixed overnight at room temperature in 2% paraformaldehyde (Fisher Scientific, Waltham, MA, AC416785000, USA). Tissues were rinsed in 1× PBS (3 × 10 min) and then heat-inactivated at 65°C for 1 h. Tissues were subsequently stained overnight in AP staining solution prepared by diluting 100× stocks of NBT (Fisher Scientific, Waltham, MA, AAJ6023006, USA) and X-PHOS (Sigma, Oakville, Canada, B6149) into AP buffer (100 mM Tris-HCl, pH 8.5, 100 mM NaCl, and 50 mM MgCl_2_) to give a final 1× concentration. After staining, the tissues were rinsed in 1× PBS (3 × 5 min) and images were captured using the Infinity 2 stereomicroscope.

### Histological analysis of SEAP expression

Representative sections of the previously stained tissues were paraffin-embedded, sectioned at 5 μm thickness and mounted onto Superfrost Plus microscope slides (Fisher Scientific, Waltham, MA, 12-1550-15, USA). The slides were deparaffinized in xylene, rehydrated through a graded ethanol series and then equilibrated in AP buffer (100 mM Tris-HCl, pH 8.5, 100 mM NaCl, and 50 mM MgCl_2_) for 5 min. The slides were stained overnight, in the dark in AP staining solution (100 mM Tris-HCl, pH 8.5, 100 mM NaCl, 50 mM MgCl2, 0.34 mg/mL nitroblue tetrazolium salt, 0.17 mg/mL X-Phos). The next day, the slides were rinsed in 1× PBS (2 × 2 min) and counterstained with nuclear fast red (Sigma, Oakville, Canada, N3020) for 1 min. The slides were rinsed in distilled water and dehydrated through a series of ethanol dips (70%, 95%, and 100%, with two dips each). Once dry, the slides were dipped in xylene (2 × 3 min), cover slipped with mounting medium (Tissue-Tek, Torrance, CA, 4583, USA) and imaged at 10× magnification using an Olympus Accu-Scope EXC-400 light microscope.

### Anti-AAV6.2FF capsid ELISA

High-binding, half-area 96-well plates (Greiner Bio-One, Kremsmünster, Austria, 0700009) were coated with 30 μL of AAV6.2FF at a concentration of 1 × 10^10^ vg per well and incubated at 4°C overnight. The next day, plates were decanted and washed three times with 100 μL of 1× PBS containing 0.2% Tween 20 (PBS-T) and blocked with 30 μL SuperBlock buffer (Thermo Fisher, Waltham, MA, 37515, USA) for 30 min at room temperature. In a separate plate, serum samples were initially diluted 1:50 in blocking buffer and serially diluted 1:2 across the plate. An ADK8 antibody (Progen Pharmaceuticals, Heidelberg, Germany, 651160) was used as a negative control and diluted the same as the serum samples in pre-selected wells. The AAV6.2FF-coated plates were decanted and the serum samples were transferred to these plates. The plates were incubated at 37°C for 1 h and then washed three times with 0.1% PBS-T. A goat anti-porcine HRP conjugated IgG antibody (Fisher Scientific, Waltham, MA, AP166PMI, USA) was diluted 1:5,000 in blocking buffer and added to each well (30 μL) and incubated at 37°C for 1 h. The secondary antibody solution was decanted and washed three times with 0.2% PBS-T. A TMB solution was prepared using a TMB substrate kit (Thermo Scientific, Waltham, MA, 34021, USA) and 30 μL was added to each well and incubated in the dark for 15 min at room temperature. Absorbance was measured at 600 nm using a Promega GloMax plate reader and the reciprocal antibody titers were defined as the highest serum dilution yielding an OD at least twice that of the mean of the negative controls.

### SEAP assays

SEAP levels in the serum were measured using the Phospha-Light SEAP Reporter Gene Assay System (Fisher Scientific, Waltham, MA, T1017, USA). Serum samples were diluted 1:3 in 1× dilution buffer and heat-inactivated at 65°C for 30 min. The samples were cooled on ice, and then 50 μL of each sample was moved to white 96-well plates (Fisher Scientific, Waltham, MA, 655075, USA). Assay buffer (50 μL/well) was added and incubated for 5 min at room temperature. Around 50 μL of reaction buffer was then added to each well, and the plates were incubated for an additional 20 min. Luminescence was measured using the Enspire multimode plate reader (PerkinElmer) with an integration time of 0.1–1 s per well.

### Hematology and clinical chemistry

Peripheral blood was collected from piglets retro-orbitally on days 0, 7, 14, 21, and 28 in both clot-activating (Fisher Scientific, Waltham, MA, 367820, USA) and sodium heparin vacutainers (Cavala Scientific, Katy, TX, 367878, USA). Serum and plasma samples were submitted to the Animal Health Laboratory (AHL) at the University of Guelph. CBCs were performed using ADVIA 2120 hematology analyzer, and clinical biochemistry panels were analyzed using Cobas 501c. All the results were interpreted using swine-specific reference intervals established by the University of Guelph AHL and reviewed by a veterinarian at the Ontario Veterinary College (OVC).

### Statistical analysis

All graphs were made and statistical analyses were performed using the GraphPad Prism 10 software (San Diego, CA, USA). Biological replicates and the mean were shown on all graphs. All error bars represent the standard deviation (SD). A one-way ANOVA and Tukey’s post hoc test were used to evaluate differences within cohorts. *p* values of <0.05 were considered significant.

## Data and code availability

All data produced and analyzed in this study are fully available and included in the published article and its supplementary files.

## Acknowledgments

This work was supported by funding from Canadian Institutes of Health research (PJT-166009), 10.13039/501100000038NSERC partnered Collaborative Health Research project (433339), The 10.13039/501100019967Ontario Lung Association (34998), 10.13039/501100000082Cystic Fibrosis Canada (3017), 10.13039/501100000038Natural Sciences and Engineering Research Council of Canada (RGPIN-2018-04737), and 10.13039/501100000094OMAFRA Alliance (UG-T2-2024-102818). N.Z. was the recipient of an Ontario Veterinary College (OVC) PhD scholarship. We thank the technical staff at the University of Guelph animal facility for their animal care services.

All animal procedures were conducted in compliance with the Canadian Council on animal care guidelines and approved by the University of Guelph Animal Care Committee under Animal Utilization Protocol #5005. A total of 14 two-week-old York/Landrace/Duroc piglets (male and female) were obtained from the Ontario Swine Research Center and enrolled in one of two different experiments: administration optimization with 3% Evans dye or dose optimization with AAV6.2FF vector. Piglets were given 2 days to acclimate prior to experimentation. After treatment, piglets were housed in social groups with environmental enrichment at the OVC Animal Isolation Unit except for six piglets used in the dye distribution experiment that were euthanized within 1 h of administration. Piglets were monitored and fed several times daily by trained personnel.

## Author contributions

Conceptualization, N.Z. and S.K.W.; methodology, N.Z., E.L.H., C.Y., B.A.Y.S., M.M.G., Y.P., D.W., A.V., L.G.A., and S.K.W.; project administration, N.Z., and S.K.W.; supervision, J.L.C., L.G.A., and S.K.W.; visualization and writing – original draft preparation, N.Z.; writing – reviewing and editing, B. Thompson, B. Thebaud, J.L.C., L.G.A., and S.K.W.; funding acquisition, B. Thebaud and S.K.W.

## Declaration of interests

S.K.W. and B. Thompson are the scientific and business founders, respectively, of Avamab Pharma Inc., a pre-clinical, pre-revenue stage company dedicated to research and development of AAV gene therapies for the treatment and prevention of infectious diseases. B. Thebaud and S.K.W. are co-founders of Inspire Biotherapeutics, a pre-clinical, pre-revenue stage company dedicated to research and development of AAV gene therapies for the treatment of monogenic lung diseases. B. Thebaud and S.K.W. are inventors on a US patent for the AAV6.2FF capsid, which is owned by the University of Guelph. This patent (US20190216949) is licensed to Avamab Pharma Inc. and Inspire Biotherapeutics. The funders had no role in the design of the study; in the collection, analyses, or interpretation of data; in the writing of the manuscript, or in the decision to publish the results.

## Supplemental information

Document S1. Figures S1–S16

Document S2. Article plus supplemental information
