# Supplementary material for: Efficient and safe lung gene delivery using AAV6.2FF in neonatal pigs demonstrates pediatric translational potential
Source: Mol Ther Adv. 2026 May 14;34(2):201739. doi: 10.1016/j.omta.2026.201739 (PMC13223870; doi:10.1016/j.omta.2026.201739)
Supplement: Document S1. Figures S1–S16 [file mmc1.pdf]

**OMTA, Volume *34***

## Supplemental information

# Efficient and safe lung gene delivery using AAV6.2FF in neonatal pigs demonstrates pediatric translational potential

**Nicole Zielinska, Erin L. Howard, Cici Yang, Brenna A.Y. Stevens, Melanie M. Goens, Yanlong Pei, Brad Thompson, Jeff L. Caswell, Bernard Thebaud, Douglas Wey, Alexander Valverde, Luis G. Arroyo, and Sarah K. Wootton**

## Supplemental Figures

A.

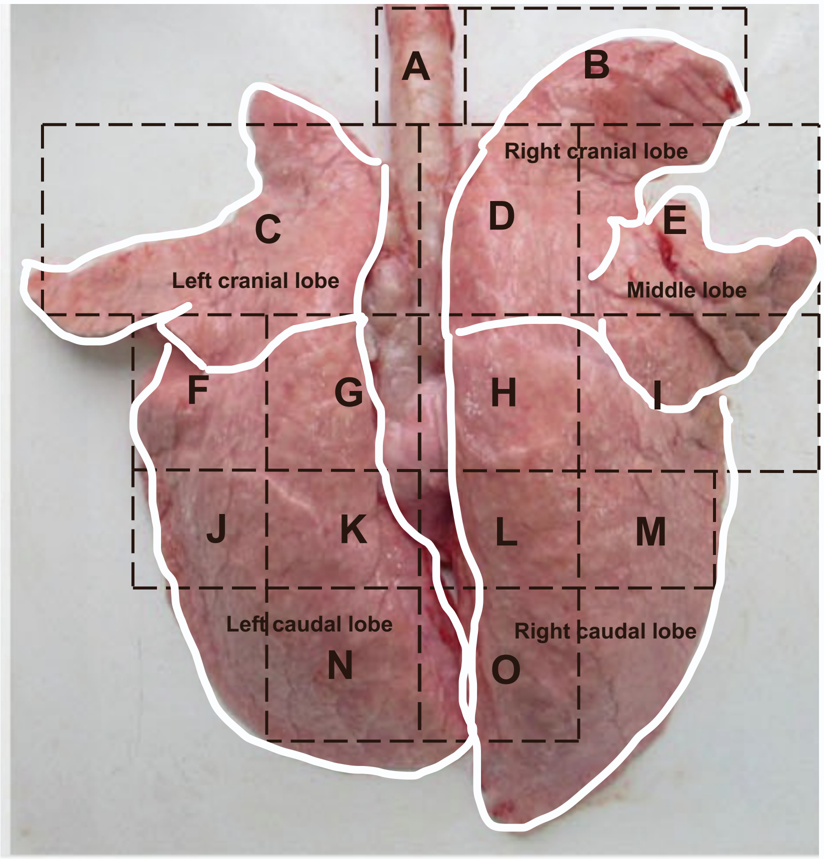


B.

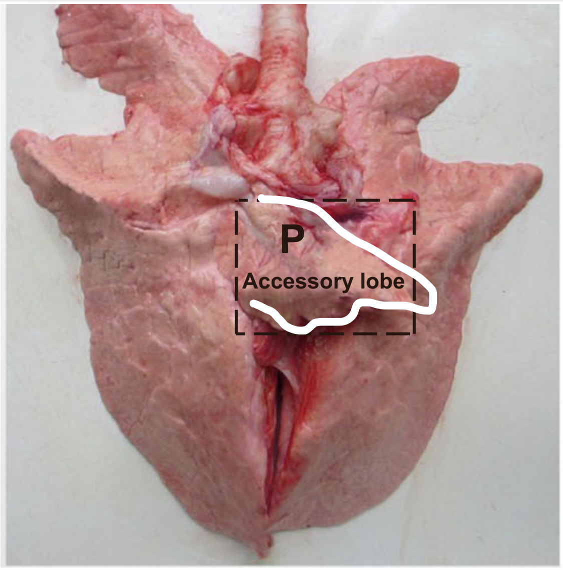

**Figure S1. Sampling sections for the neonatal pig lung.** Following AAV administration, piglets were euthanized, and the lungs were harvested en bloc for downstream analysis. The lungs were divided into 16 sampling regions, labelled A-P, to allow for a clearer picture of the transgene distribution across specific anatomical sites. (A) sampling sections for A through O from the dorsal view and (B) sampling section P from a ventral view. Figures adapted from Establishment of a Model of Mycoplasma hyopneumoniae infection using Bama miniature pigs by Gan et al (https://doi.org/10.1186/s43014-020-00034-w) used under https://creativecommons.org/licenses/by/4.0/. Image was modified by adding region labels.

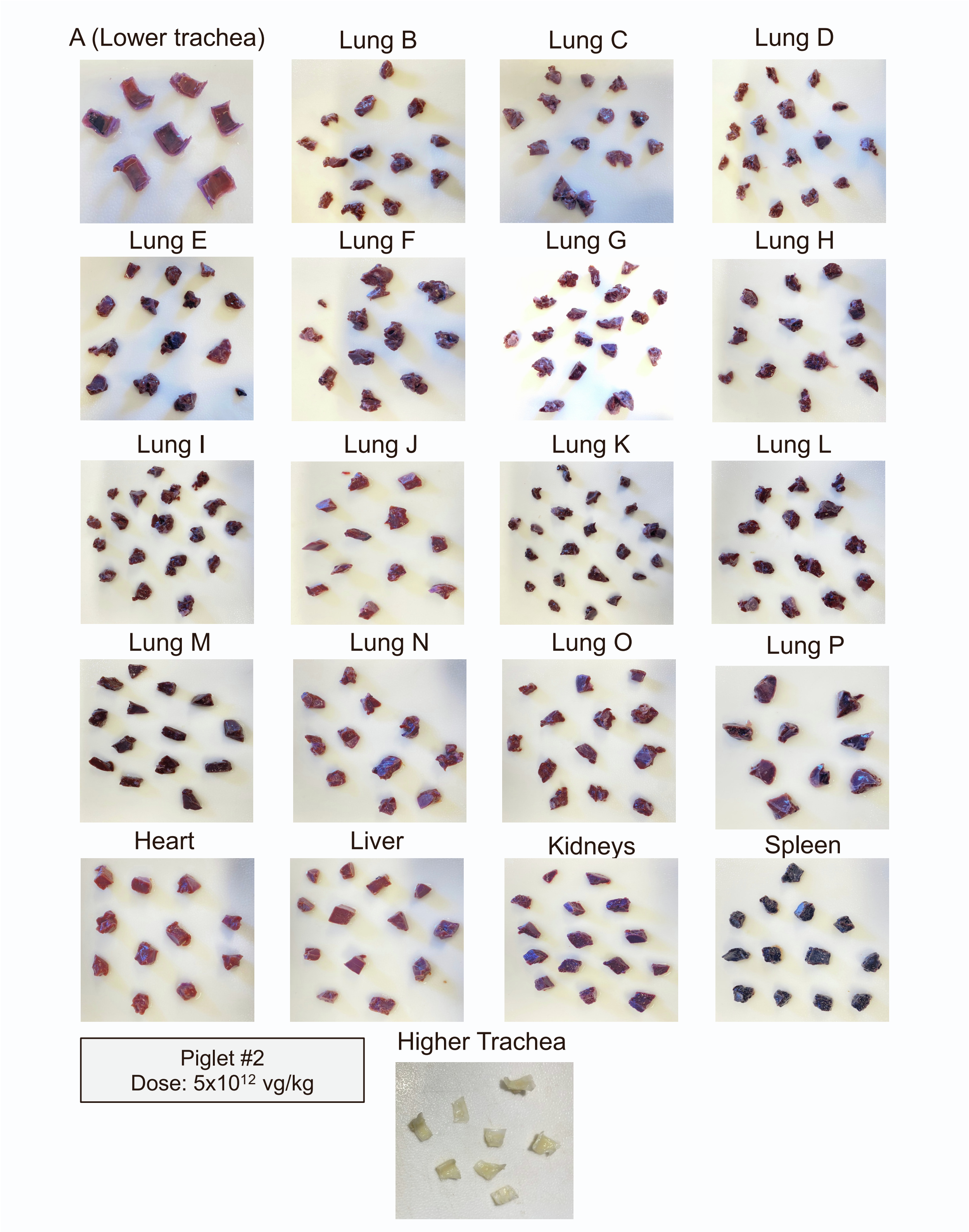
A (Lower trachea)
Lung B
Lung C
Lung D
Lung E
Lung F
Lung G
Lung H
Lung I
Lung J
Lung K
Lung L
Lung M
Lung N
Lung O
Lung P
Heart
Liver
Kidneys
Spleen
Piglet #2
Dose: 5x10^12 vg/kg
Higher Trachea

**Figure S2. Subdivided tissue sampling for detailed mapping of alkaline phosphatase expression in piglet 2.** The lungs from piglet #2 ($5x10^{12}$ vg/kg AAV6.2FF-CASI-SEAP) were divided into 16 sampling sites (A-P), and each region was further subdivided into smaller fragments before alkaline phosphatase staining. Non-pulmonary tissues were arbitrarily sectioned and stained for alkaline phosphatase. Shown are the individually stained fragments from all lung sections (A-P), the heart, liver, kidneys, spleen, and the higher trachea.

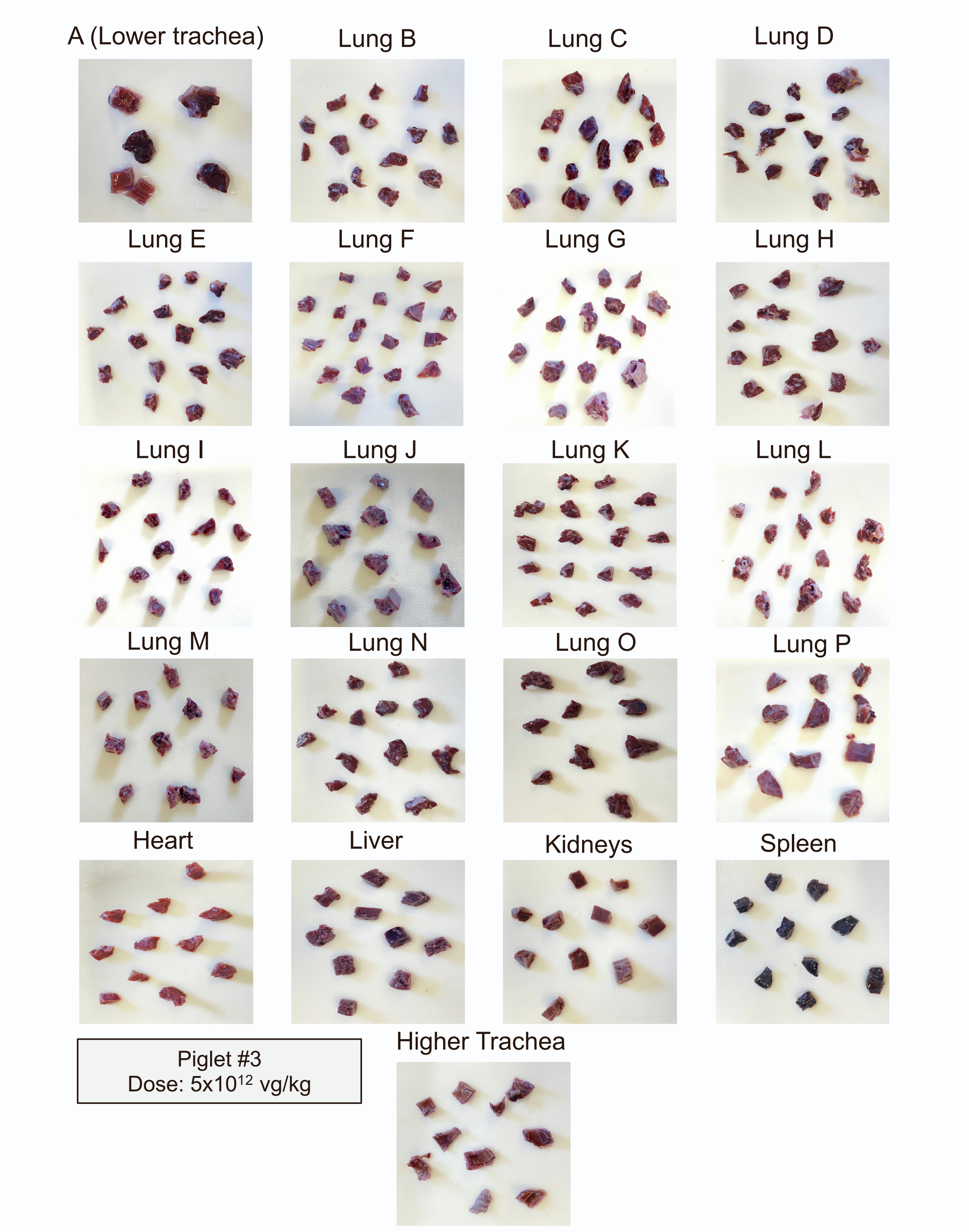
A (Lower trachea)
Lung B
Lung C
Lung D
Lung E
Lung F
Lung G
Lung H
Lung I
Lung J
Lung K
Lung L
Lung M
Lung N
Lung O
Lung P
Heart
Liver
Kidneys
Spleen
Piglet #3
Dose: 5x10^12 vg/kg
Higher Trachea

**Figure S3. Subdivided tissue sampling for detailed mapping of alkaline phosphatase expression in piglet 3.** The lungs from piglet #3 ($5x10^{12}$ vg/kg AAV6.2FF-CASI-SEAP) were divided into 16 sampling sites (A-P), and each region was further subdivided into smaller fragments before alkaline phosphatase staining. Non-pulmonary tissues were arbitrarily sectioned and stained for alkaline phosphatase. Shown are the individually stained fragments from all lung sections (A-P), the heart, liver, kidneys, spleen, and the higher trachea.

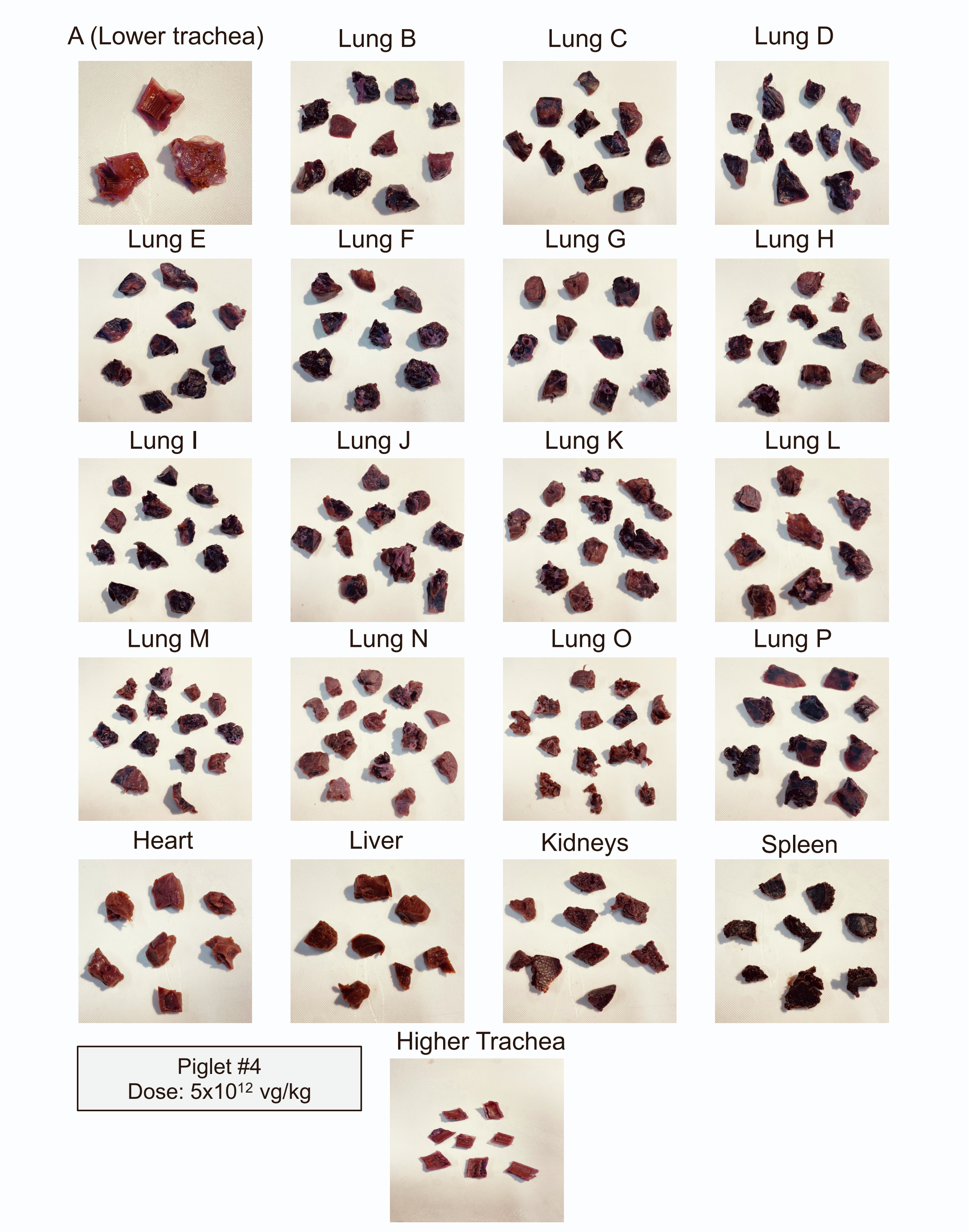
A (Lower trachea)
Lung B
Lung C
Lung D
Lung E
Lung F
Lung G
Lung H
Lung I
Lung J
Lung K
Lung L
Lung M
Lung N
Lung O
Lung P
Heart
Liver
Kidneys
Spleen
Piglet #4
Dose: 5x10^12 vg/kg
Higher Trachea

**Figure S4. Subdivided tissue sampling for detailed mapping of alkaline phosphatase expression in piglet 4.** The lungs from piglet #4 ($1.73 \times 10^{13}$ vg/kg AAV6.2FF-CASI-SEAP) were divided into 16 sampling sites (A-P), and each region was further subdivided into smaller fragments before alkaline phosphatase staining. Non-pulmonary tissues were arbitrarily sectioned and stained for alkaline phosphatase. Shown are the individually stained fragments from all lung sections (A-P), the heart, liver, kidneys, spleen, and the higher trachea.

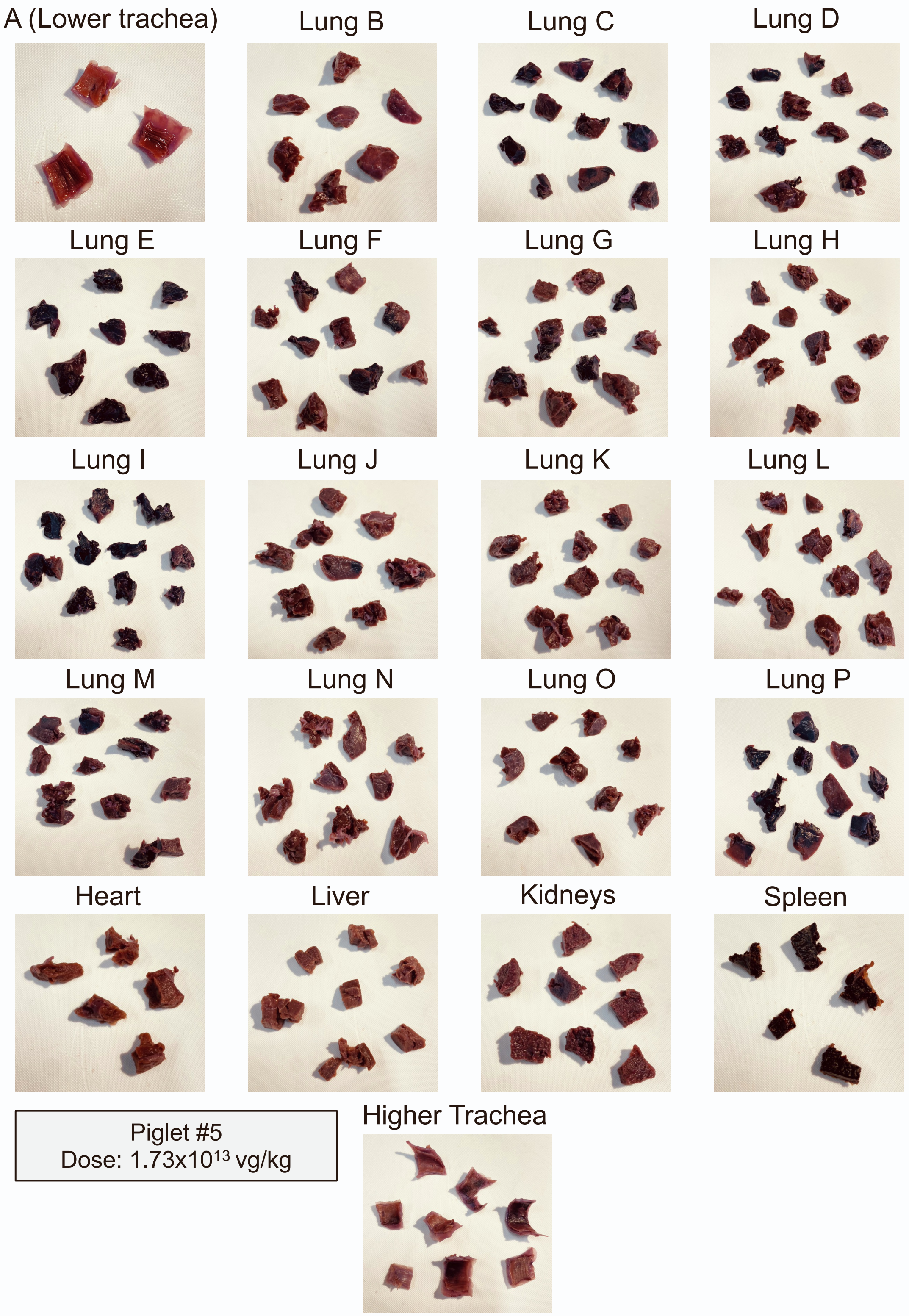
A (Lower trachea)
Lung B
Lung C
Lung D
Lung E
Lung F
Lung G
Lung H
Lung I
Lung J
Lung K
Lung L
Lung M
Lung N
Lung O
Lung P
Heart
Liver
Kidneys
Spleen
Piglet #5
Dose: 1.73x10^13 vg/kg
Higher Trachea

**Figure S5. Subdivided tissue sampling for detailed mapping of alkaline phosphatase expression in piglet 5.** The lungs from piglet #5 ($1.73x10^{13}$ vg/kg AAV6.2FF-CASI-SEAP) were divided into 16 sampling sites (A-P), and each region was further subdivided into smaller fragments before alkaline phosphatase staining. Non-pulmonary tissues were arbitrarily sectioned and stained for alkaline phosphatase. Shown are the individually stained fragments from all lung sections (A-P), the heart, liver, kidneys, spleen, and the higher trachea.

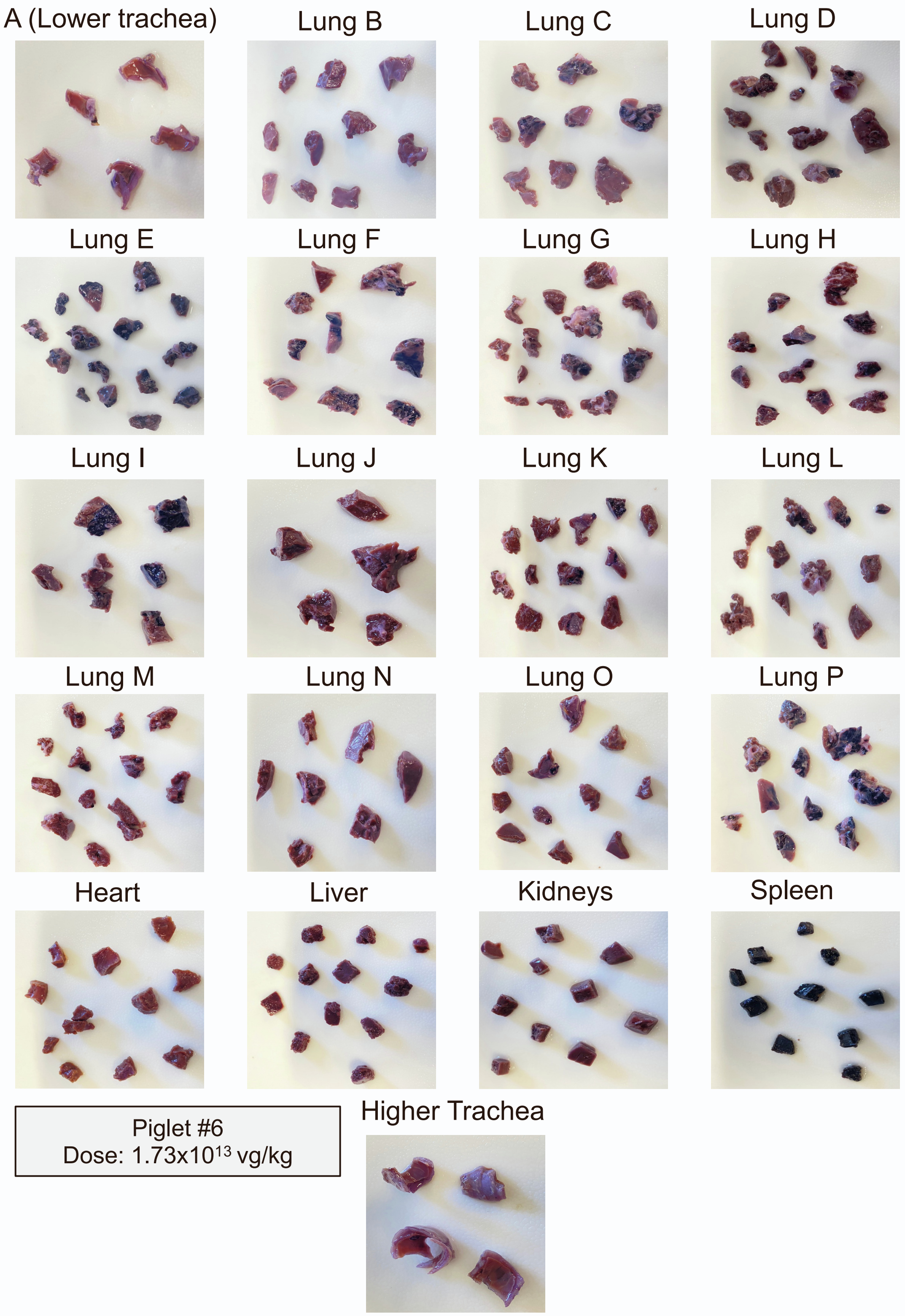
A (Lower trachea)
Lung B
Lung C
Lung D
Lung E
Lung F
Lung G
Lung H
Lung I
Lung J
Lung K
Lung L
Lung M
Lung N
Lung O
Lung P
Heart
Liver
Kidneys
Spleen
Piglet #6
Dose: 1.73x10$^{13}$ vg/kg
Higher Trachea

**Figure S6. Subdivided tissue sampling for detailed mapping of alkaline phosphatase expression in piglet 6.** The lungs from piglet #6 ($1.73x10^{13}$ vg/kg AAV6.2FF-CASI-SEAP) were divided into 16 sampling sites (A-P), and each region was further subdivided into smaller fragments before alkaline phosphatase staining. Non-pulmonary tissues were arbitrarily sectioned and stained for alkaline phosphatase. Shown are the individually stained fragments from all lung sections (A-P), the heart, liver, kidneys, spleen, and the higher trachea.

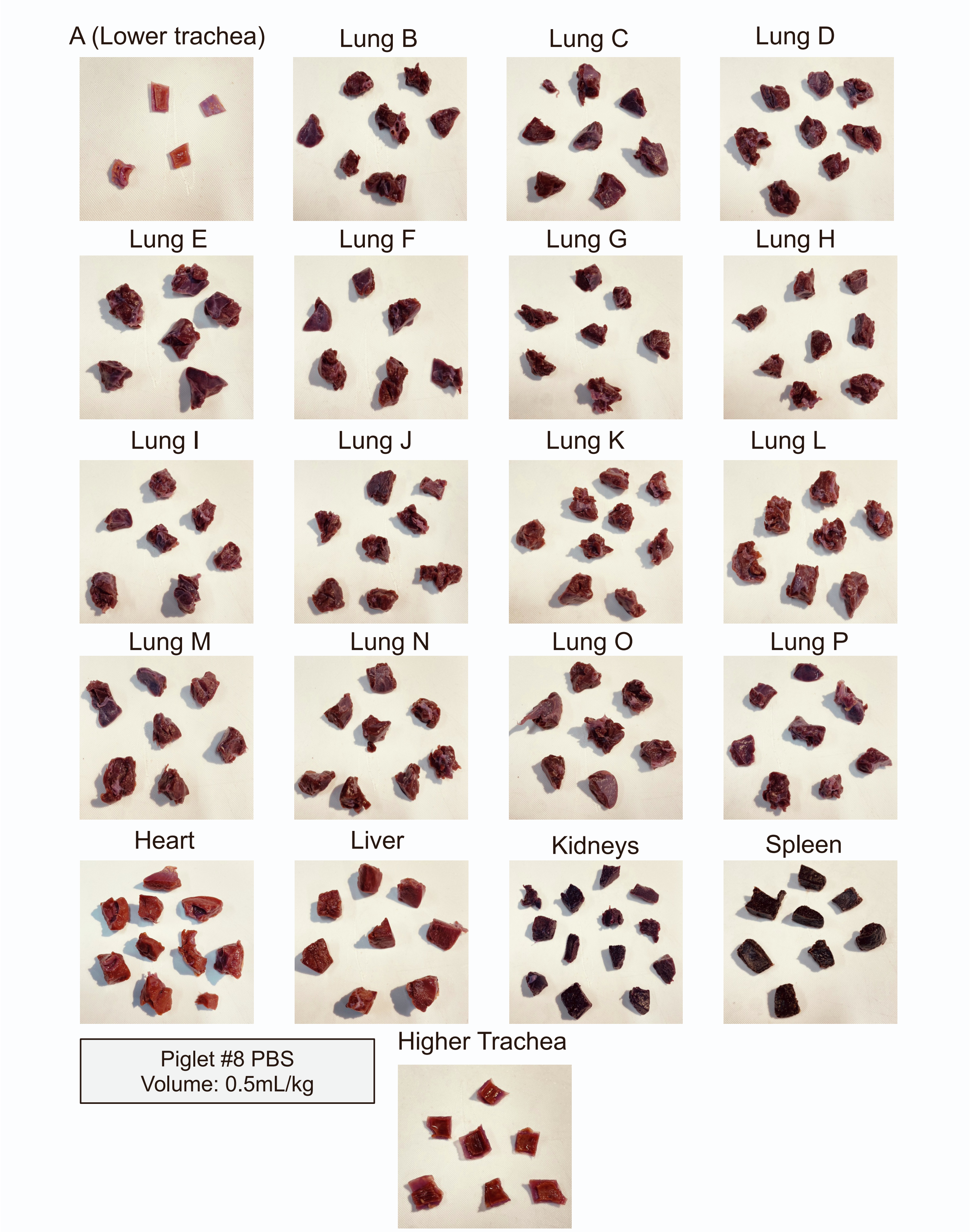
A (Lower trachea)
Lung B
Lung C
Lung D
Lung E
Lung F
Lung G
Lung H
Lung I
Lung J
Lung K
Lung L
Lung M
Lung N
Lung O
Lung P
Heart
Liver
Kidneys
Spleen
Piglet #8 PBS
Volume: 0.5mL/kg
Higher Trachea

**Figure S7. Subdivided tissue sampling for detailed mapping of alkaline phosphatase expression in piglet 8.** The lungs from piglet #8 (0.5 mL/kg PBS) were divided into 16 sampling sites (A-P), and each region was further subdivided into smaller fragments before alkaline phosphatase staining. Non-pulmonary tissues were arbitrarily sectioned and stained for alkaline phosphatase. Shown are the individually stained fragments from all lung sections (A-P), the heart, liver, kidneys, spleen, and the higher trachea.

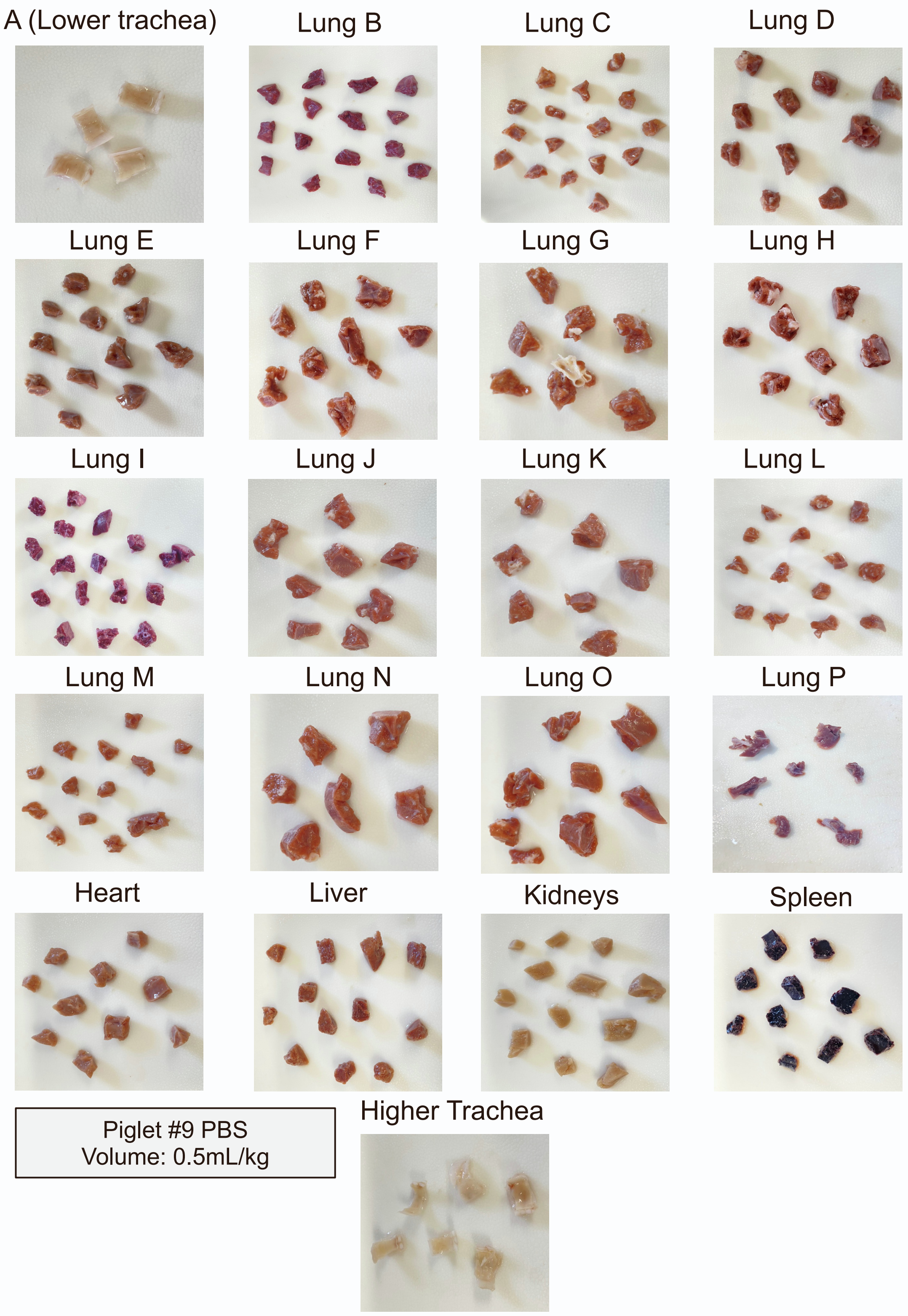
A (Lower trachea)
Lung B
Lung C
Lung D
Lung E
Lung F
Lung G
Lung H
Lung I
Lung J
Lung K
Lung L
Lung M
Lung N
Lung O
Lung P
Heart
Liver
Kidneys
Spleen
Piglet #9 PBS
Volume: 0.5mL/kg
Higher Trachea

**Figure S8. Subdivided tissue sampling for detailed mapping of alkaline phosphatase expression in piglet 9.** The lungs from piglet #9 (0.5 mL/kg PBS) were divided into 16 sampling sites (A-P), and each region was further subdivided into smaller fragments before alkaline phosphatase staining. Non-pulmonary tissues were arbitrarily sectioned and stained for alkaline phosphatase. Shown are the individually stained fragments from all lung sections (A-P), the heart, liver, kidneys, spleen, and the higher trachea.

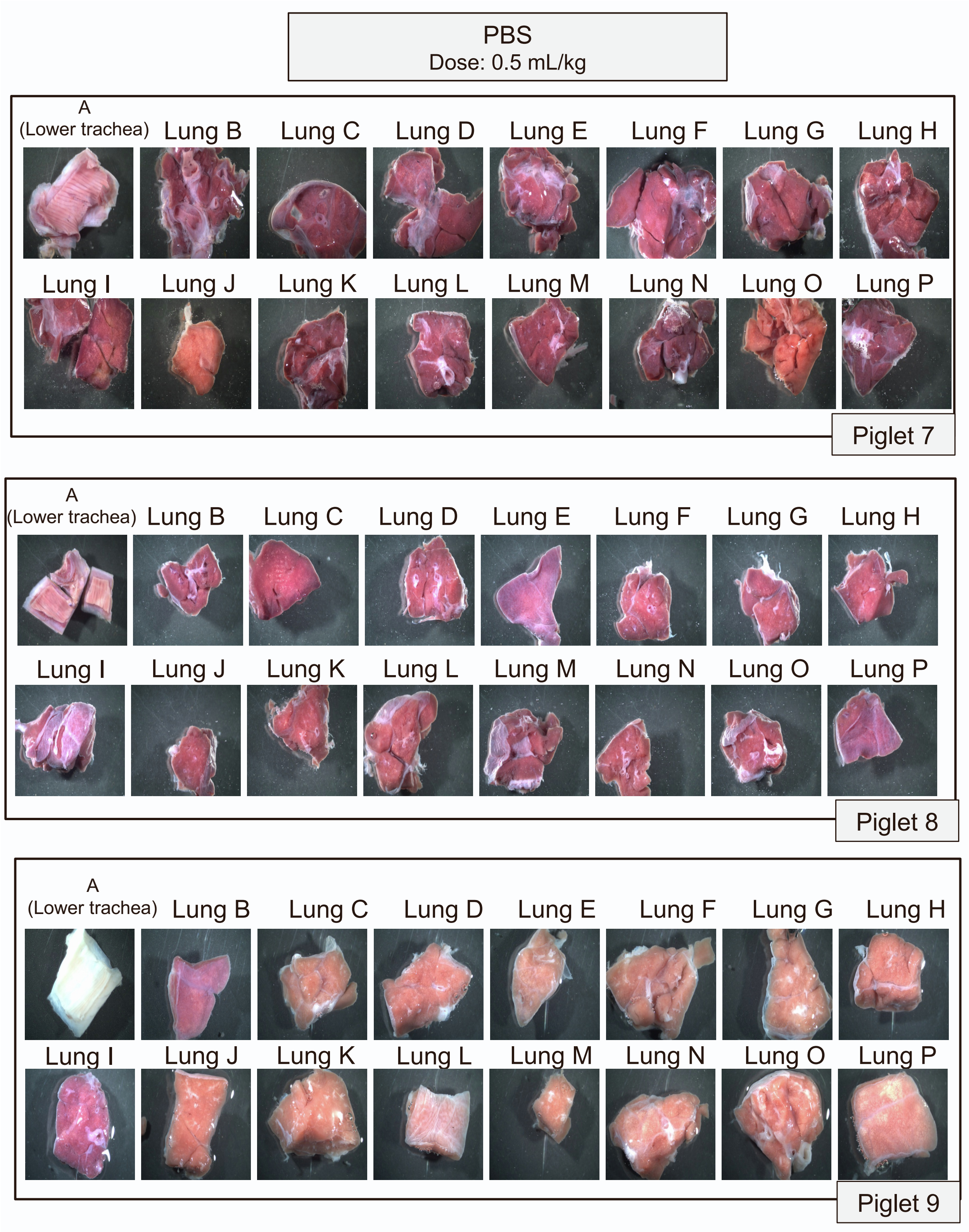
PBS
Dose: 0.5 mL/kg
A
(Lower trachea)
Lung B
Lung C
Lung D
Lung E
Lung F
Lung G
Lung H
Lung I
Lung J
Lung K
Lung L
Lung M
Lung N
Lung O
Lung P
Piglet 7
A
(Lower trachea)
Lung B
Lung C
Lung D
Lung E
Lung F
Lung G
Lung H
Lung I
Lung J
Lung K
Lung L
Lung M
Lung N
Lung O
Lung P
Piglet 8
A
(Lower trachea)
Lung B
Lung C
Lung D
Lung E
Lung F
Lung G
Lung H
Lung I
Lung J
Lung K
Lung L
Lung M
Lung N
Lung O
Lung P
Piglet 9

**Figure S9. Macroscopic evaluation of AP-stained negative control lung sections following PBS administration in neonatal piglets.** 2-week-old piglets (n=3) were administered 0.5 mL/kg of PBS via endotracheal atomization. After 28 days, piglets were euthanized, and the lungs were collected en bloc. Each lung was divided into 16 representative sampling regions (A-P), fixed, and stained for alkaline phosphatase. Shown are representative macroscopic images of the stained lung sections from the three individual piglets.

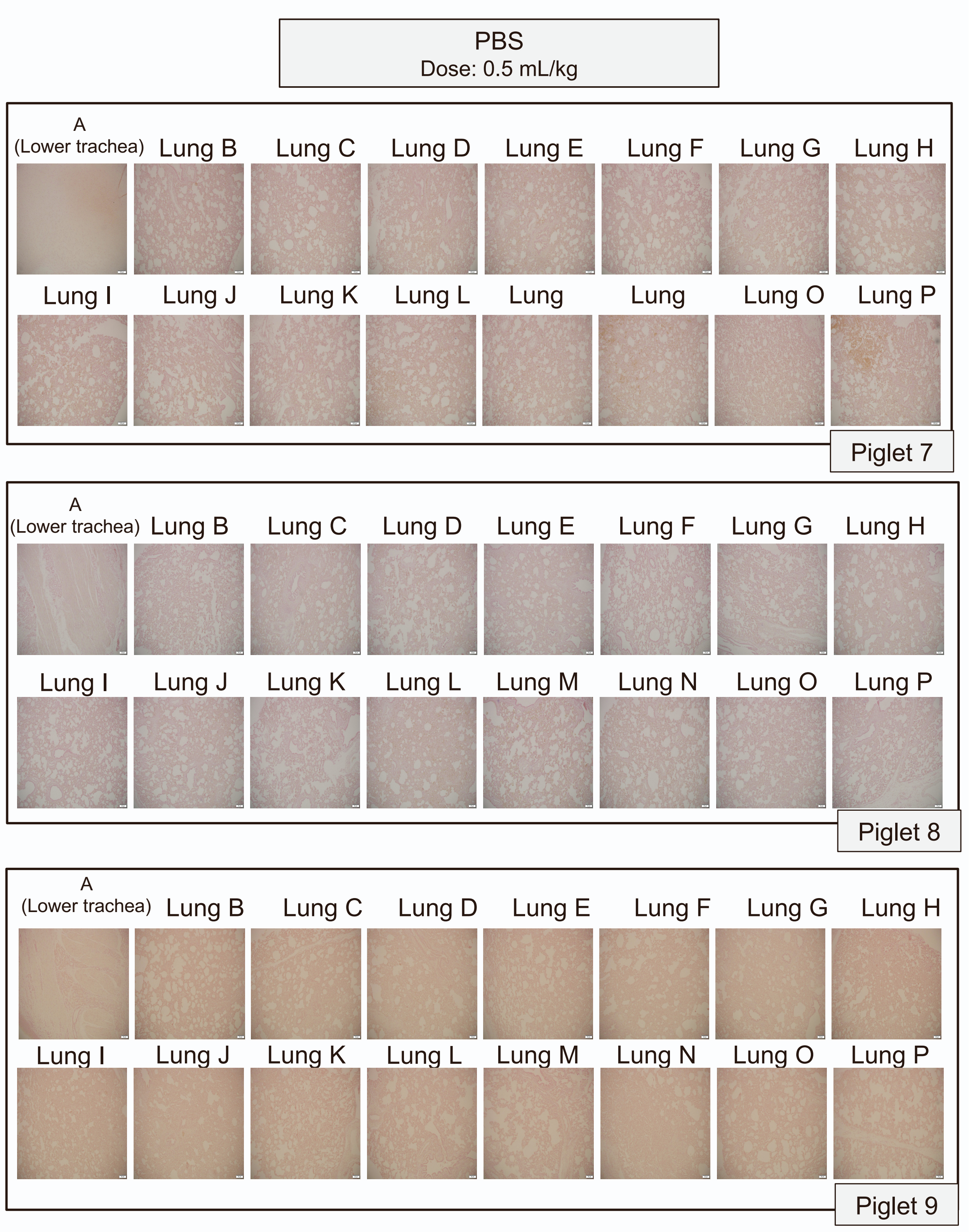
PBS
Dose: 0.5 mL/kg
A
(Lower trachea)
Lung B
Lung C
Lung D
Lung E
Lung F
Lung G
Lung H
Lung I
Lung J
Lung K
Lung L
Lung
Lung
Lung O
Lung P
Piglet 7
A
(Lower trachea)
Lung B
Lung C
Lung D
Lung E
Lung F
Lung G
Lung H
Lung I
Lung J
Lung K
Lung L
Lung M
Lung N
Lung O
Lung P
Piglet 8
A
(Lower trachea)
Lung B
Lung C
Lung D
Lung E
Lung F
Lung G
Lung H
Lung I
Lung J
Lung K
Lung L
Lung M
Lung N
Lung O
Lung P
Piglet 9

**Figure S10. Histological evaluation of AP-stained negative lung sections following PBS administration in neonatal piglets.** 2-week-old piglets (n=3) were administered 0.5 mL/kg of PBS via endotracheal atomization and euthanized 28 days later. Lungs were excised, sectioned into 16 representative sampling regions (A-P), fixed and stained for alkaline phosphatase activity. Representative tissue sections from each region were paraffin-embedded, sectioned at 5 μm and stained for alkaline phosphatase, followed by nuclear fast red counterstain. Images were taken at 10x magnification. Shown are the histological pictures from all 16 representative lung sections in each of the three piglets.

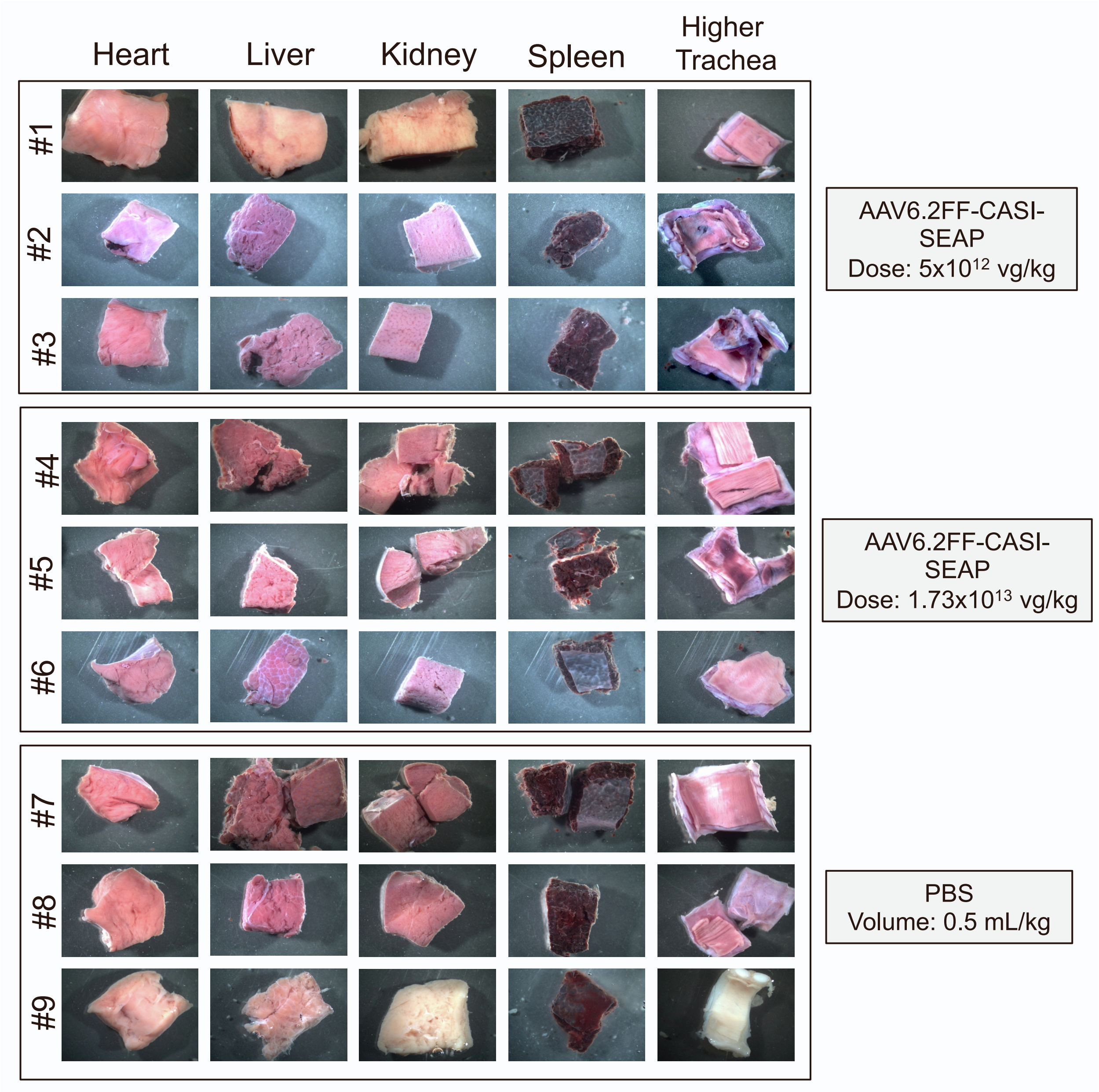


**Figure S11. A macroscopic view of AP-stained non-pulmonary tissues from all treated piglets.** 28 days after pigs received either AAV6.2FF-CASI-SEAP or PBS, non-pulmonary tissues including the heart, liver, kidney, spleen and higher trachea, were collected from all piglets. Each tissue was further divided into sections, fixed in 2% paraformaldehyde, heat-inactivated and stained for alkaline phosphatase. Representative images from each tissue and piglet are shown.

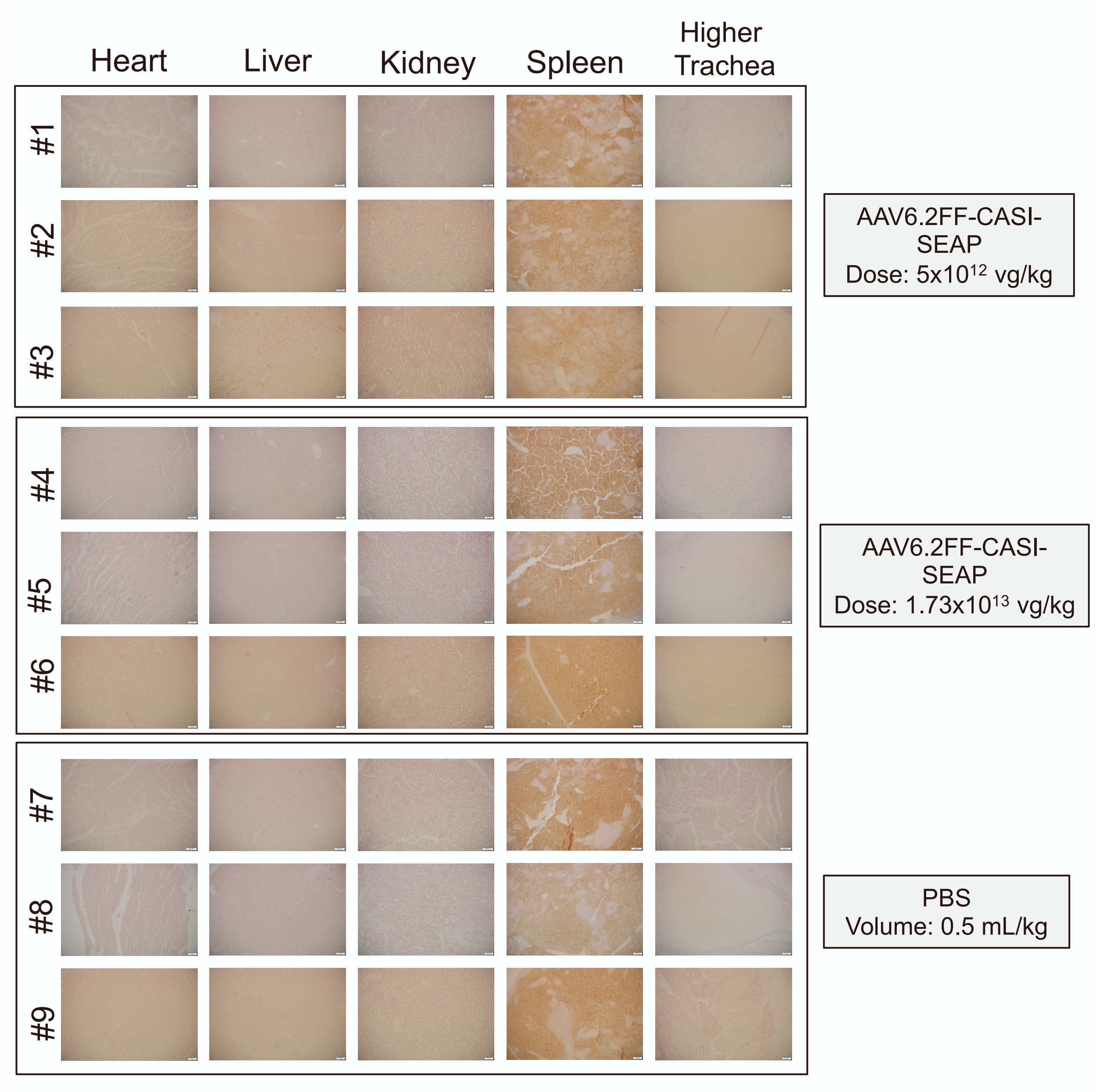


**Figure S12. A microscopic view of AP-stained non-pulmonary tissues from all treated piglets.** 28 days after pigs received either AAV6.2FF-CASI-SEAP or PBS, non-pulmonary tissues including the heart, liver, kidney, spleen and higher trachea, were collected from all piglets. Representative tissue sections previously stained for alkaline phosphatase (AP) were paraffin-embedded, sectioned, re-stained for AP, and counterstained with nuclear fast red. Images were taken at 10x magnification. Shown are the representative histological sections of non-pulmonary tissues collected from all nine pigs.

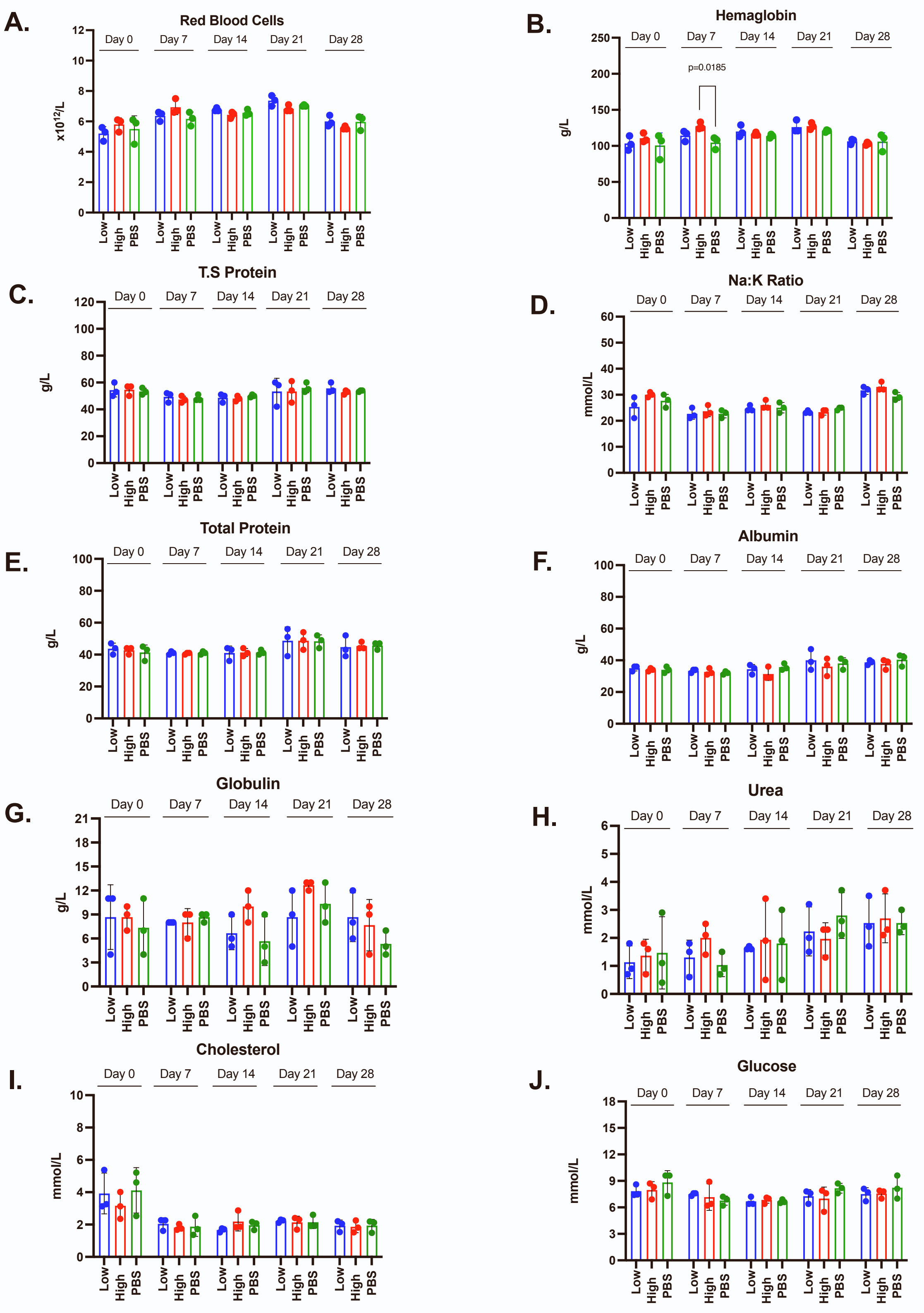

A.
Red Blood Cells
x10^12/L
Day 0
Day 7
Day 14
Day 21
Day 28
Low
High
PBS
B.
Hemaglobin
g/L
p=0.0185
C.
T.S Protein
g/L
D.
Na:K Ratio
mmol/L
E.
Total Protein
g/L
F.
Albumin
g/L
G.
Globulin
g/L
H.
Urea
mmol/L
I.
Cholesterol
mmol/L
J.
Glucose
mmol/L

**Figure S13. Safety and tolerability assessment of AAV6.2FF-CASI-SEAP administration via hematology and biochemistry profiling.** Piglets were administered either a low dose of AAV6.2FF-CASI-SEAP ($5x10^{12}$ vg/kg) (n=3), a high dose ($1.73x10^{13}$ vg/kg) (n=3), or 0.5 mL/kg (n=3) of PBS. Blood was collected from piglets at days 0, 7, 14, 21, and 28 post-treatments for hematological (A) red blood cells, (B) hemoglobin, (C) total serum (T.S) protein, and biochemistry analysis (D) $Na^{+}/K^{+}$ ratio, (E) total protein, (F) albumin, (G) globulin, (H) urea, (I) cholesterol, (J) glucose. Mean values are shown on all graphs, with error bars representing the standard deviation (SD). A one-way ANOVA with Tukey's post hoc test was used to assess significance between treatment groups at each time point**.**

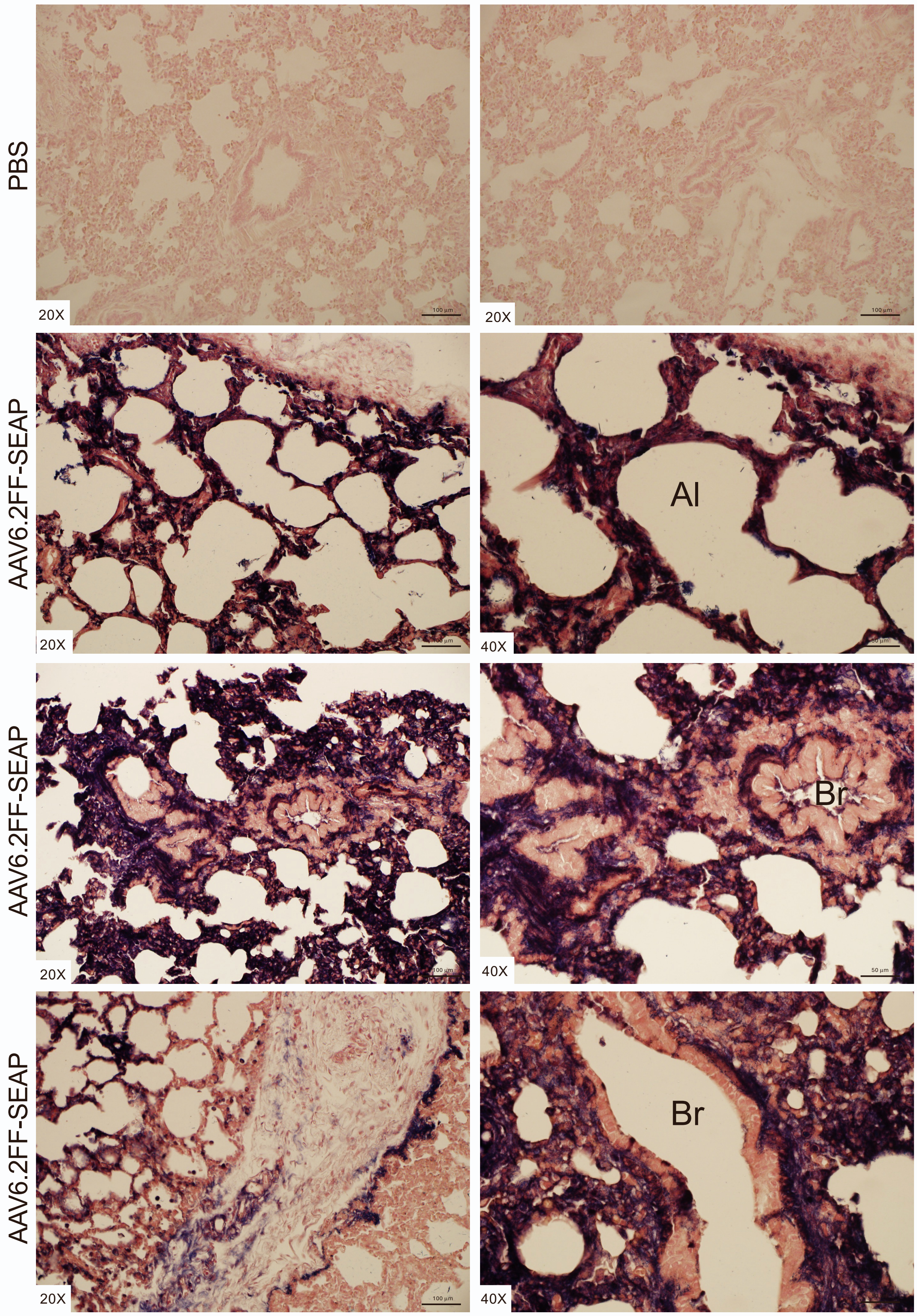


**Figure S14. High-magnification analysis of SEAP expression in pig lung tissue following AAV6.2FF-SEAP administration.** Higher-magnification images of lung sections from pigs administered phosphate-buffered saline (PBS) or AAV6.2FF-SEAP were stained for secreted alkaline phosphatase (SEAP) expression. IHC staining revealed robust SEAP signal within the alveolar regions (Al) of lungs from AAV6.2FF-SEAP–treated pigs, whereas little to no SEAP staining was observed in cells lining the bronchial airways (Br). PBS-treated lung sections showed no detectable SEAP staining. This staining pattern is consistent with preferential transduction of alveolar type II cells, the target cell population for therapeutic intervention in surfactant protein B deficiency.

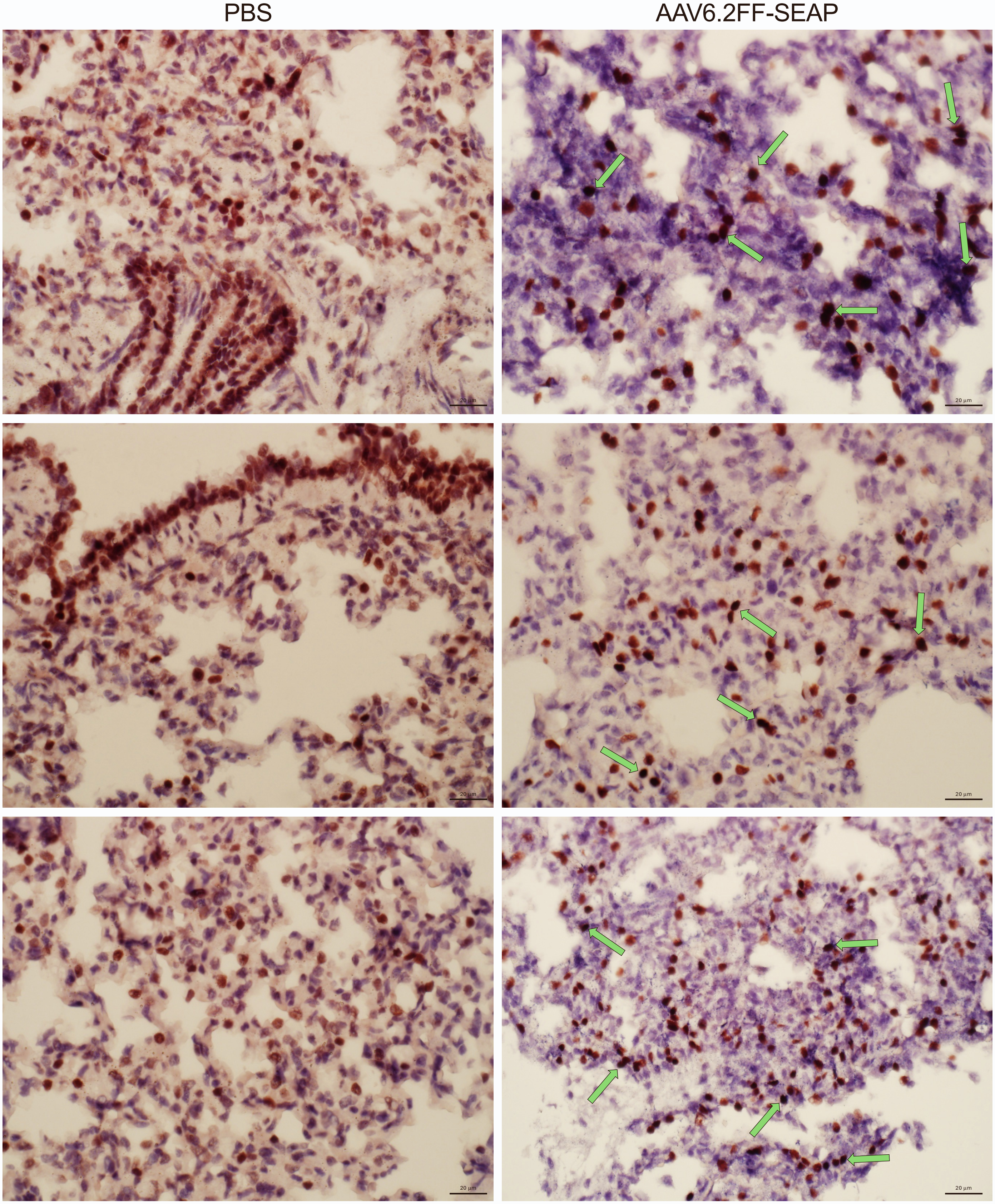


**Figure S15. Immunohistochemical detection of epithelial marker TTF-1 in pig lung sections following AAV6.2FF-SEAP administration.** Lung sections were obtained from pigs administered phosphate-buffered saline (PBS) or AAV6.2FF-SEAP. Sections were stained for secreted alkaline phosphatase (SEAP) expression, followed by immunohistochemical (IHC) staining for the epithelial marker thyroid transcription factor-1 (TTF-1). Representative images were acquired at 60× magnification. TTF-1 staining was detected throughout lung sections from both PBS- and AAV6.2FF-SEAP–treated pigs. In lung sections from AAV6.2FF-SEAP–treated pigs, cells positive for both SEAP and TTF-1 were observed throughout the tissue. Green arrows indicate representative double-positive cells expressing both SEAP and TTF-1.

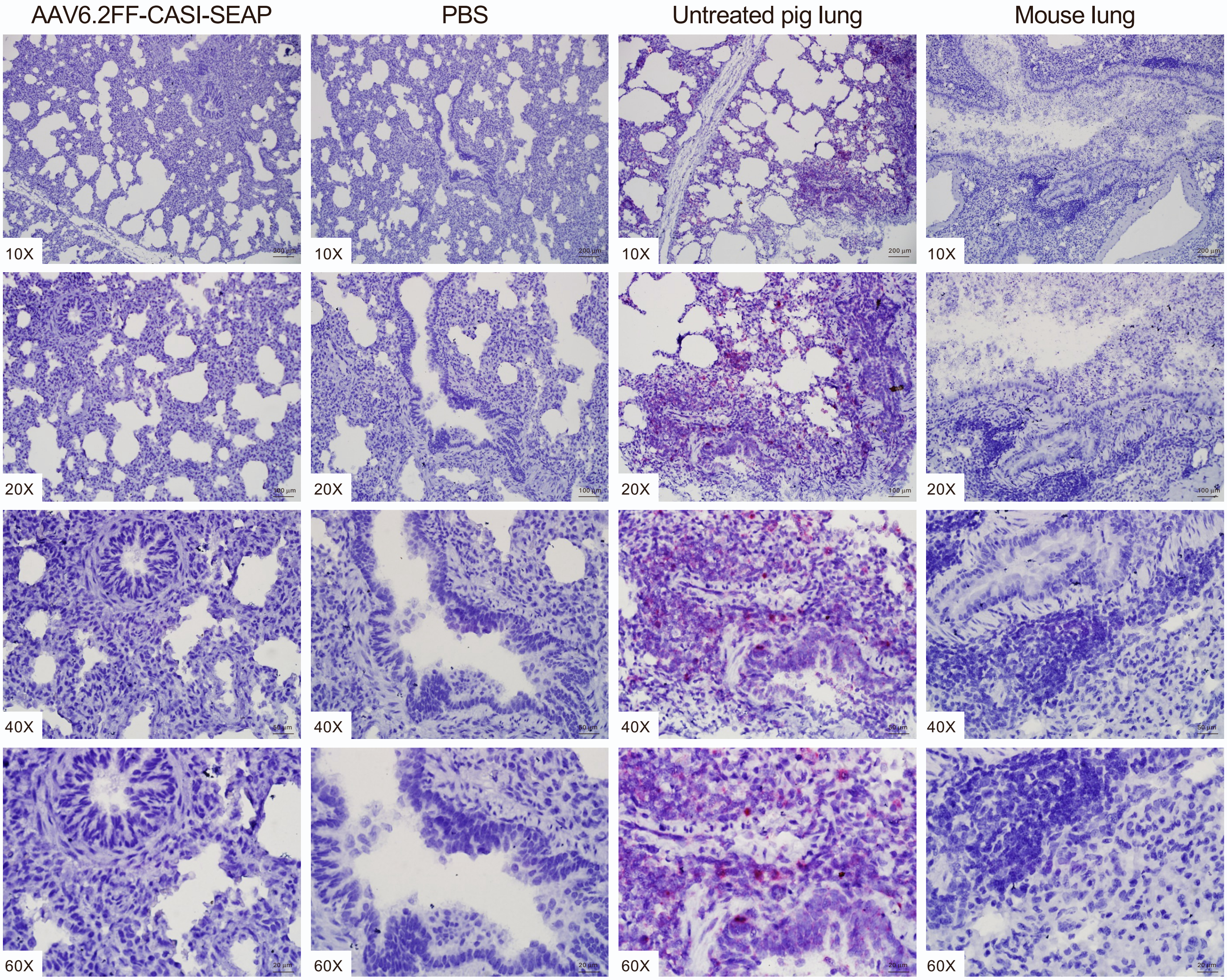


**Figure S16**. **Assessment of RNAscope compatibility in porcine lung tissues following heat inactivation and SEAP histochemical staining.** Lung sections were obtained from pigs administered phosphate-buffered saline (PBS) or AAV6.2FF-SEAP intratracheally, as well as from untreated pigs. PBS- and AAV6.2FF-SEAP–treated lungs were fixed, subjected to heat inactivation at 65 °C for 1 h, stained for secreted alkaline phosphatase (SEAP), and subsequently paraffin embedded. Control porcine lung tissue was harvested fresh, immediately fixed, paraffin embedded, and sectioned. Mouse lung tissue was included as an additional control. Tissue sections (4 µm) were deparaffinized and rehydrated, then subjected to antigen retrieval for 15 min using the antigen retrieval buffer provided with the RNAscope kit. To confirm tissue amenability to RNAscope following heat inactivation and SEAP staining, the RNAscope 2.5 High-Definition RED Assay (Advanced Cell Diagnostics; cat. no. 322350) was performed. Sections were subjected to manual antigen retrieval at 95 °C for 15 min and hybridized with a commercial porcine housekeeping gene probe (Pig PP1B positive control; cat. no. 428591). Signal amplification was conducted using the HybEZ oven according to the manufacturer's instructions, with detection using Fast Red chromogen and hematoxylin counterstaining. Slides were examined to assess signal distribution and imaged using a Nikon Digital Sight 10 camera and CellSens imaging software. Robust RNAscope signal was detected in fresh, untreated pig lung tissue, confirming assay functionality; however, no detectable signal was observed in lung tissues subjected to heat inactivation and SEAP staining. These findings indicate that RNAscope is not compatible with the heat-inactivated, SEAP-stained lung tissues and therefore is not a viable approach for identifying the cell types transduced by the AAV6.2FF vector under these experimental conditions.
